# Solid-Phase Oligosaccharide Synthesis with Highly Complexed Peptidoglycan Fragments

**DOI:** 10.3390/molecules30132787

**Published:** 2025-06-28

**Authors:** Yuichiro Kadonaga, Ning Wang, Atsushi Shimoyama, Yukari Fujimoto, Koichi Fukase

**Affiliations:** 1 Division of Science, Institute for Radiation Sciences, The University of Osaka, 1-1 Machikaneyama, Toyonaka 560-0043, Osaka, Japan; kadonagay19@irs.osaka-u.ac.jp; 2 State Key Laboratory of Biopharmaceutical Preparation and Delivery, Institute of Process Engineering, University of Chinese Academy of Sciences, 1 North 2nd Street, Zhongguancun, Haidian District, Beijing 100190, China; wangning@mail.ipe.ac.cn; 3 Department of Chemistry, Graduate School of Science, The University of Osaka, 1-1 Machikaneyama, Toyonaka 560-0043, Osaka, Japan; ashimo@chem.sci.osaka-u.ac.jp; 4 Department of Chemistry, Faculty of Science and Technology, Keio University, 3-14-1 Hiyoshi, Kohoku-ku, Yokohama 223-8522, Kanagawa, Japan

**Keywords:** solid-phase oligosaccharide synthesis, peptidoglycan, glycosylation, innate immunity

## Abstract

Peptidoglycan (PGN) is a component of bacterial cell walls; its fragments are recognized by the cytoplasmic receptors Nod1 and Nod2, thereby promoting the production of inflammatory cytokines and antibodies. To further elucidate these biological defense mechanisms, a large and stable supply of the PGN fragments via chemical synthesis is essential. However, the synthesis and purification of long PGN fragments are quite challenging due to their low solubility. In this study, we efficiently synthesized PGN fragments via solid-phase oligosaccharide synthesis (SPOS). Using the JandaJel™ Wang resin (JJ-Wang), an octasaccharide glycan chain of PGN was constructed by repeating glycosylation reactions to elongate β-1,4-linked disaccharide units composed of MurNAc and GlcNAc. To enhance reactivity, glycosylation was performed in a mixed solvent comprising C_4_F_9_OEt/CH_2_Cl_2_/THF with the intention of promoting substrate concentration onto the solid support through the fluorophobic effect, affording the PGN octasaccharide in a 19% overall yield (10 steps). Subsequently, after deprotection of the *O*-Fmoc, *N*-Troc, and ethyl ester groups, *N*- and *O*-acetylation proceeded smoothly, owing to the high swelling property of JJ-Wang. Peptide condensation with L-Ala-D-isoGln(OBn) and carboxylic acids was also achieved. Finally, cleavage of the PGN fragment from the resin with TFA afforded the desired octasaccharide with dipeptides in a 2.3% overall yield (15 steps).

## 1. Introduction

Solid-phase synthesis is a method for constructing molecules on inorganic or organic insoluble supports such as silica gel, alumina, and organic polymers. This technique is widely employed in peptide synthesis because it enables the stepwise construction of molecular backbones using sequential reactions with building blocks on a solid support, offering rapid synthesis without purification. In particular, the use of solid-phase peptide synthesizers enables efficient synthesis of long-chain peptides consisting of several tens of residues.

Solid-phase oligosaccharide synthesis (SPOS) is gaining increasing attention, particularly for the synthesis of glycans with repeating structures. Nicolaou et al. synthesized an elicitor-type dodecasaccharide using an *o*-nitrobenzyloxy benzoyl linker on a polystyrene resin, achieving a 10% yield over 10 steps [1,2]. Seeberger et al. made a major breakthrough in SPOS by developing the “Automated Solid-Phase Synthesis” system [3], enabling the efficient synthesis of long-chain glycans. Notably, they achieved the automated solid-phase synthesis of α-1,6-polymannosides comprising up to 100 monosaccharide units and also succeeded in constructing branched glycans containing 151 sugar units [4]. The contribution of Seeberger to the development of SPOS is of high significance, with his recent reports being outstanding, including SPOS of C1-functionalized and algal fucoidan oligosaccharides [5,6,7]. Furthermore, his development of automated synthesis equipment with integrated microwave irradiation has substantially reduced SPOS reaction times [8].

Boons et al. achieved α-selective glycosylation on solid support using an (S)-(phenylthiomethyl)benzyl group at the C2 position [9], where the formation of a *trans*-decalin-like intermediate facilitated axial attack by nucleophilic reagents, affording the α-glycoside in 25% yield (13 steps).

Codee et al. reported automated SPOS of β-mannuronic acid alginates [10]. They achieved 1,2-*cis*-β-selective glycosylation with a mannuronic acid donor to produce oligosaccharides in excellent yields: tetrasaccharide (47%), octasaccharide (20%), and dodecasaccharide (11%). Subsequent hydrogenation yielded β-mannuronic acid alginates, major components of algal cell walls. Furthermore, they reported an automated SPOS of oligorhamnans, which form a backbone of the bacteria cell wall of group A *Streptococcus* [11]. For α-selective glycosylation with dirhamnoside on the solid support, they employed cyanopivaloyl (PivCN) ester as a neighboring group, successfully synthesizing hexadecarhamnoside.

Demchenko et al. achieved SOPS of *N*-linked glycoprotein core glycans with β-selective mannosylation via hydrogen-bond-mediated aglycone delivery (HAD) of the picoloyl group [12]. They also developed an automated SPOS using a high-performance liquid chromatography (HPLC) system [13,14]. HPLC autosamplers can enable highly efficient investigation of key reactions, including glycosylation on the solid support. By using this system, SPOS of several oligosaccharides with [1→4] and [1→6] linkages was achieved.

Furthermore, our group reported SPOS of complex *N*-glycans [15]; using the JandaJel™ resin, the octasaccharide of *N*-glycan was successfully constructed in a 27% yield (11 steps). Such results highlight the utility of SPOS for the divergent and efficient synthesis of long-chain oligosaccharides. However, there are few reports on SPOS of glycoconjugates, such as glycopeptides and glycolipids, which is still under development [16,17].

**Figure 1:**
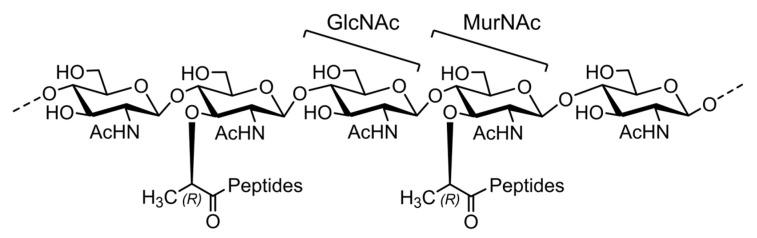
Structure of the bacterial cell wall peptidoglycan. GlcNAc: *N*-Acetyl glucosamine, MurNAc: *N*-Acetylmuramic acid.

Peptidoglycan (PGN), which is a major component of bacterial cell walls, has a repeating β-1,4-linkage structure of MurNAc and GlcNAc coupled with long peptide chains at the lactamide moiety of muramic acid to form three-dimensional bridge structures (Figure 1). Biologically, the PGN fragments promote the production of inflammatory cytokines and antibodies by binding to proteins, including the innate immune receptors Nod1 and Nod2, peptidoglycan recognition proteins (PGRPs), and other PGN-recognizing enzymes and lectins [18,19,20,21,22,23,24,25,26]. To further elucidate their biological functions, a stable supply of single and highly pure PGN fragments is essential.

**Figure 2:**
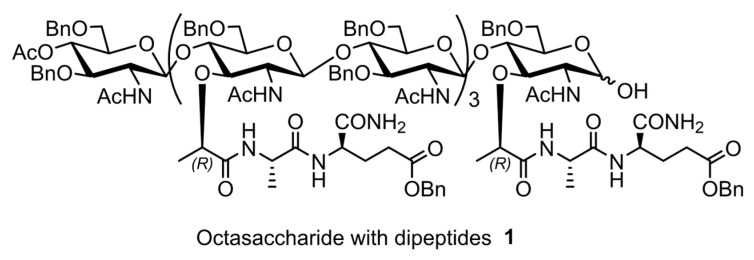
Structure of octasaccharide with four dipeptide moieties **1**.

We previously developed a method for the synthesis and evaluation of various types of PGN fragments [27,28,29,30,31,32,33,34,35,36,37]. PGN fragments of various lengths, including octasaccharides, were synthesized by 2 + 2 and 4 + 4 glycosylation. Moreover, di-, tri-, tetra-, penta-, and hepta-peptides were condensed into their PGN fragments. However, the purification of longer PGN fragments is challenging due to their low solubility. Furthermore, their reduced reactivity and increased number of reactive sites often require additional reaction and purification steps. To overcome this challenge, we focused on SPOS. In 2014, we reported the first successful SPOS of a PGN tetrasaccharide with two dipeptide moieties [38], demonstrating both glycan elongation and peptide condensation onto multiple carboxylic acid cites of PGN fragments on a solid phase, thus expanding the scope of SPOS. In this study, we demonstrate the SPOS of PGN octasaccharide with four dipeptide moieties **1**, based on detailed investigations of glycosylation, capping, and peptide coupling, with the aim of further extending the glycan chain and introducing additional dipeptide units. (Figure 2).

## 2. Results and Discussion

**Scheme 1:**
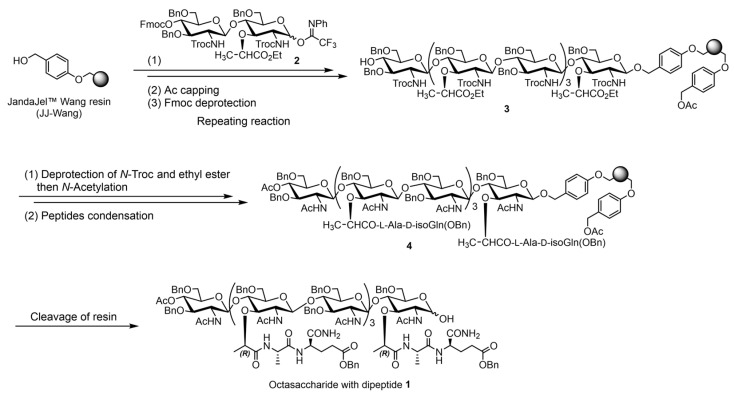
Synthetic strategy of octasaccharide with dipeptides **1**.

The synthetic strategy for the PGN fragments is shown in Scheme 1. The JandaJel™ Wang resin (JJ-Wang) was employed as a solid-phase support, exhibiting an ether-crosslinked polystyrene structure to ensure swelling [12,13,15,39,40,41,42]. A disaccharide composed of a muramyl and a glucosaminyl residue **2** was used as a glycosyl donor, with *N*-phenyl trifluoroacetimidate as a PGN repeating unit. The *N*-phenyl-trifluoroacetimidates developed by Yu enable glycosylation under relatively mild conditions [28,43] and are more suitable as donor substrates for less reactive acceptors on the resin than the trichloroacetimidate used in Schmidt glycosylation [36,44]. After the glycosylation reaction, the unreacted hydroxy groups were capped with acetyl groups. Subsequently, the Fmoc group was removed to expose a free hydroxy group as the next reactive site. By repeating glycosylation, acetyl capping, and Fmoc deprotection, octasaccharide **3** was obtained. The *N*-Troc groups were deprotected along with the ethyl esters and subsequently converted into *N*-Ac groups. The dipeptides were then condensed with the MurNAc moieties to obtain compound **4** on the resin. Finally, cleavage from the resin yielded the desired octasaccharide with dipeptides **1**.

As reported in our previous study [38], glycosylation was conducted with donor **5** and TMSOTf at −15 °C on the JJ-Wang resin, affording the monosaccharide-loaded resin **6** in a moderate yield. In this study, the glycosylation conditions were further optimized; under optimal conditions, approximately 45% of the reactive sites of the resin were accessible (Table 1). Investigation of the optimal conditions involved using the JJ-Wang resin (50.0 mg: 1.0 mmol/g) with donor **5** (1.0 eq.) and TMSOTf (0.5 eq.) in CH_2_Cl_2_ at −15 °C for 1 h, affording **6** in a 22% yield (Entry 1). Increasing the amount of donor **5** to 2.0 and 3.0 eq. improved the yield to 35% and 45%, respectively (Entries 2 and 3). However, even with 3.0 eq. of **5**, the loading of muramyl monosaccharide remained suboptimal. This low efficiency was attributed to the undesired cleavage of the *p*-oxybenzyl linker on JJ-Wang in the presence of TMSOTf (Scheme S5). To mitigate this, THF was introduced to reduce the Lewis acidity of TMSOTf, increasing the yield to 34% (Entry 4). Aiming to further improve the loading ratio, a mixture of CH_2_Cl_2_/THF (9:1) was employed, and glycosylation with donor **5** (1.0 eq.) at −15 °C for 1 h improved the yield to 44% (Entry 5). Extending the reaction time did not further enhance loading (Entry 6), and further yield improvement appeared limited due to steric hindrance near the *p*-oxybenzyl alcohol moiety (reactive functional group on the resin). Although the Wang ChemMatrix^®^ resin (composed of flexible polyethylene glycol) offered better swelling and reduced steric hindrance, it afforded lower yields, possibly due to sequestration of TMSOTf by the ether groups on the resin (Scheme S2). Because the reactivity of free hydroxy groups on the polymer is heterogeneous, it is preferable not to introduce glycans at the less reactive hydroxy sites, as this can be advantageous for subsequent glycan chain elongation. Based on these findings, full glycosylation of all *p*-oxybenzyl groups was not pursued. Instead, the conditions of Entry 5 were selected as optimal for efficient synthesis.

**Scheme 2:**
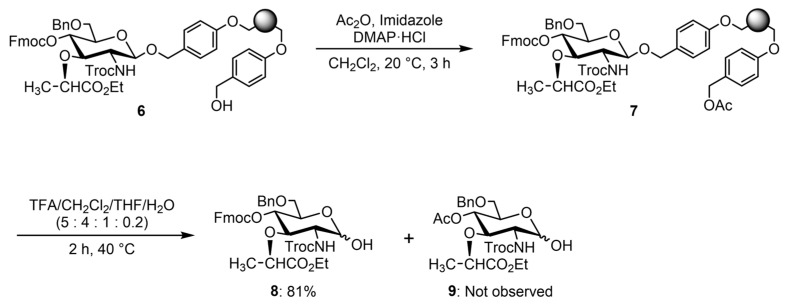
Ac capping and cleavage from resin.

After loading the muramyl monosaccharide, unreacted hydroxy groups of **6** were capped with acetyl groups to prevent the formation of immature PGN units (Scheme 2). However, the protection of the hydroxy groups under basic conditions such as Ac_2_O/Pyridine/CH_2_Cl_2_ (1:1:1) led to undesired deprotection of the Fmoc group and acetylation (Scheme S3). Ac capping under neutral conditions was, therefore, employed, using imidazole and DMAP·HCl as the nucleophilic catalysts under base-free conditions [45]. The cleavage of the muramyl monosaccharide from the resin was conducted in a mixture of TFA/CH_2_Cl_2_/THF/H_2_O as previously reported [38]. As expected, **8** was obtained in an 81% yield without formation of the acetylated byproduct **9**.

**Scheme 3:**
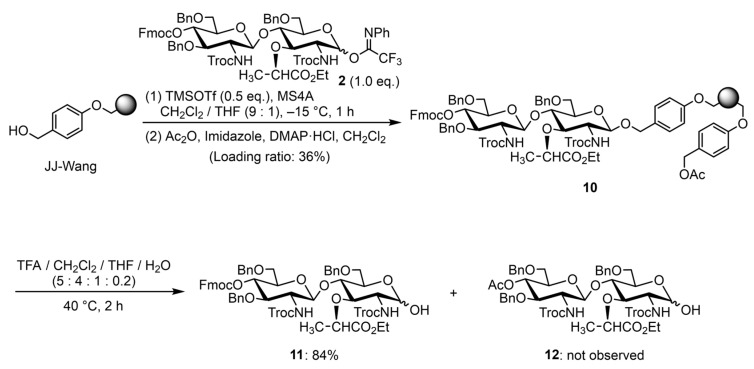
Introduction of disaccharide **2**, Ac capping, and cleavage from resin.

The synthesis of the glycan chain of PGN containing muramyl and glucosaminyl disaccharides was examined under the optimized conditions (Scheme 3). First, the glycosylation was performed using donor **2** (1.0 eq.) and TMSOTf (0.5 eq.) in a CH_2_Cl_2_/THF mixed solvent at −15 °C for 1 h, resulting in 36% of the hydroxyl groups on JJ-Wang reacting with **2**. Although the molecular weight of the donor was increased by more than 1.5 times, the loading efficiency onto the resin was maintained at a comparable level. After Ac capping of the free hydroxy groups with Ac_2_O (160.0 eq.), imidazole (20.0 eq.), and DMAP·HCl (5.0 eq.), disaccharide **11** was cleaved from the resin and obtained in an 84% yield without formation of the acetylated byproduct **12**.

**Scheme 4:**
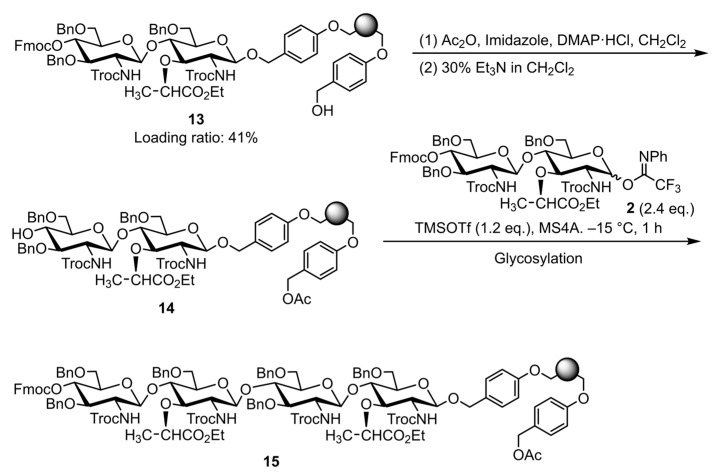
Synthesis of the solid-supported tetrasaccharide **15**.

After obtaining disaccharide-loaded JJ-Wang resin 13 (41% loading), we investigated the elongation of the glycan chain (Scheme 4). Following acetyl capping and Fmoc deprotection of 13, acceptor 14 was glycosylated with donor **2** (2.4 equiv.) and TMSOTf (1.2 equiv.) to afford tetrasaccharide 15 (Table 2). However, the glycosylation yield was unsatisfactory at 56% (Entry 1).

The reactivity of functional groups on a polymer is often heterogeneous, depending on their local environment. In our system, we hypothesized that such variability, along with steric hindrance around the hydroxy groups, contributed to the reduced glycosylation efficiency. To address this issue, we introduced the fluorous solvent C_4_F_9_OEt. Due to its low polarity and the fluorophobic effect characteristic of fluorous solvents, C_4_F_9_OEt does not readily penetrate the ether-bridged structure of the JJ-Wang resin. Instead, the glycosyl donor is expected to accumulate within the resin matrix, swollen by CH_2_Cl_2_ and THF, owing to the fluorophobic effect, thereby facilitating its interaction with hydroxy groups. We previously demonstrated that highly efficient glycosylation using CH_2_Cl_2_/C_4_F_9_OEt enabled the synthesis of complex *N*-glycans [15], and we anticipated a similar enhancement effect in this system. Nonetheless, the 2 + 2 glycosylation yield remained modest at 61% [38].

We hypothesized that the glycosylation under harsh conditions (CH_2_Cl_2_/C_4_F_9_OEt/THF (9:10:1) for 3 h at 0 °C) led to the undesired cleavage of the *p*-oxybenzyl linker on JJ-Wang, prompting us to re-optimize the solvent composition, reaction time, and temperature. Using a modified solvent system of CH_2_Cl_2_/C_4_F_9_OEt/THF (5:4:1) at −15 °C for 1 h, to JJ-Wang, we achieved a significantly improved glycosylation yield of 83% (Entry 2). These optimized conditions are expected to facilitate further elongation of the glycan chain.

**Scheme 5:**
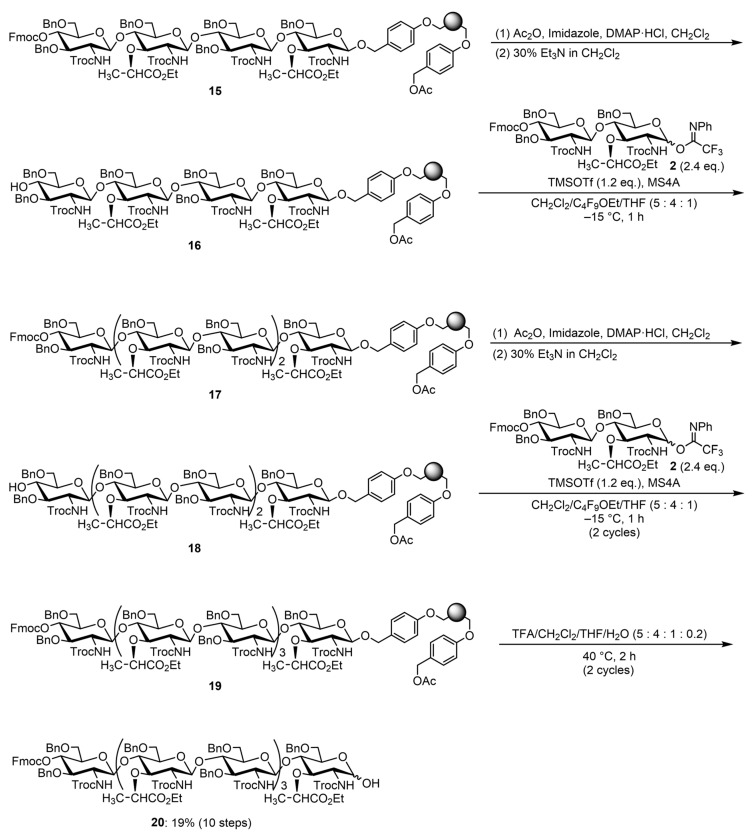
Synthesis of octasaccharide **20**.

**Figure 3:**
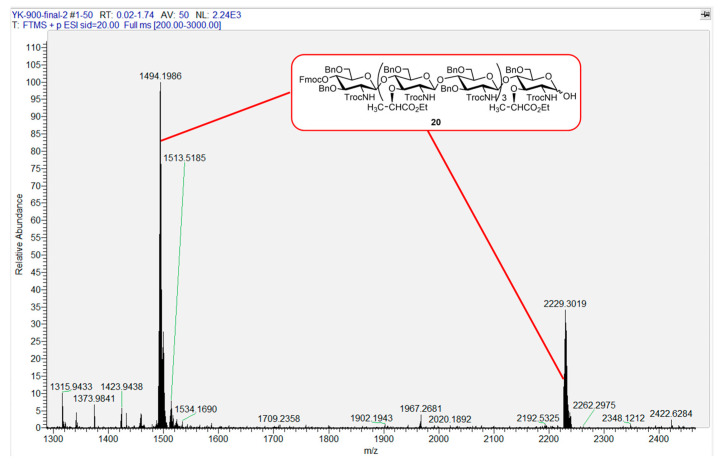
MS spectra of octasaccharide **20**.

The synthesis of the PGN octasaccharide **20** was then examined (Scheme 5). Ac capping of **15** followed by Fmoc deprotection was conducted, and subsequent glycosylation with **2** under the optimized conditions (Table 2, Entry 2) yielded the hexasaccharide with a loading yield of 68%. The production of the hexasaccharide was confirmed via ESI-LIT-Orbitrap MS analysis after cleavage of **17** from the resin with TFA/CH_2_Cl_2_/THF/H_2_O (5:4:1:0.2). Similarly, the unreacted glycosyl acceptor on the resin underwent Ac capping. Glycosylation was performed under identical conditions using the glycosyl acceptor **18** obtained after Fmoc deprotection. However, a single glycosylation did not afford a satisfactory loading yield. Therefore, the reaction was repeated twice, resulting in the formation of the octasaccharide **19** with a loading yield of 76%. The synthesized solid-supported octasaccharide **19** was shaken with TFA/CH_2_Cl_2_/THF/H_2_O (5:4:1:0.2) at 40 °C for 2 h (2 cycles), and the reaction mixture was diluted and collected. After quenching and extraction, the MS peaks of the desired octasaccharide **20** were confirmed (Figure S1). Although partially constructed di-, tetra-, and hexa-saccharides were also observed, they were removed by silica gel and LH-20 column chromatography (Figure 3), affording pure **20** in 19% yield (10 steps, starting from **13**, average yield > 84%).

**Scheme 6:**
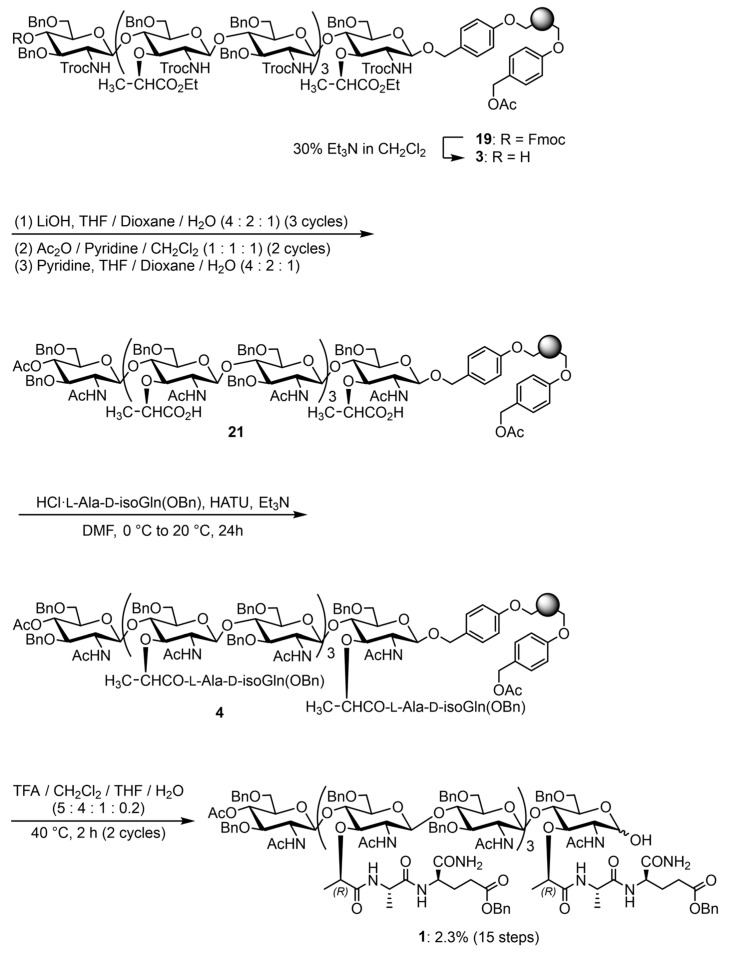
Synthesis of octasaccharide with dipeptides **1**.

The synthesis of the PGN octasaccharide with dipeptides **1** was then examined (Scheme 6). Fmoc deprotection of **19** was conducted using 30% Et_3_N in CH_2_Cl_2_, followed by hydrolysis of the muramic acid ethyl esters and *N*-Troc groups using LiOH in THF/dioxane/H_2_O (4:2:1) at 20 °C for 12 h (3 cycles) [46]. In the solution-phase synthesis of PGN, the deprotection steps involving *N*-Troc deprotection with Zn/Cu and ethyl ester hydrolysis with LiOH had to be conducted separately due to the difficulty in separating and re-deprotecting incomplete reaction products. In contrast, on the solid support, deprotection can be performed continuously without the need for purification. The liberated amino and hydroxyl groups were then acetylated with Ac_2_O/pyridine/CH_2_Cl_2_ (1:1:1) at 20 °C for 12 h (two cycles). Under these conditions, the free carboxylic acids may form mixed acetic anhydrides. Therefore, hydrolysis was performed using pyridine in THF/dioxane/H_2_O (4:2:1) at 20 °C for 24 h to yield the octasaccharide **21** with free carboxyl groups. Peptide condensation of **21** with dipeptides was performed using an excess amount of HCl·L-Ala-D-*iso*-Gln(OBn), HATU, and triethylamine in DMF at 0 to 20 °C for 24 h to obtain the PGN octasaccharide with dipeptides **4**. Finally, cleavage of **4** was performed with TFA/CH_2_Cl_2_/THF/H_2_O (5:4:1:0.2) at 40 °C for 2 h (two cycles). After extraction of the cleaved reaction mixture with CHCl_3_/MeOH (20:1), short silica gel column chromatography and HPLC purification afforded the desired octasaccharide with dipeptide **1** in a 2.3% yield (15 steps, starting from **13,** average yield > 77%). Moreover, the hydrogenation of **1** with Pd/C at 2 MPa was also successful, as confirmed by analysis via high resolution mass spectrometry (HRMS) of the deprotected product (Scheme S10).

## 3. Materials and Methods

### 3.1. Preparation of the Glycosyl Donors

The glycosyl donors **2** and **5** were synthesized from D-(+)-glucosamine hydrochloride (Tokyo Chemical Industry Co., Ltd., Tokyo, Japan) according to a previously reported method [37] and were stored in the fridge.

### 3.2. SPOS of PGN Fragments

SPOS was performed on tens of μmol or less scale. PGN fragments were synthesized on the solid support (see Supplementary Materials) via glycosylation at −15 °C for 1 h using the glycosyl donor, TMSOTf, and MS4A. Ac capping was performed under neutral conditions with Ac_2_O, imidazole, and DMAP·HCl in CH_2_Cl_2_. Fmoc deprotection was conducted with 30% Et_3_N in CH_2_Cl_2_. Hydrolysis of *N*-Troc and ethyl ester was performed with LiOH in THF/dioxane/H_2_O (4:2:1), while *N*- and *O*-acetylation was conducted with Ac_2_O/pyridine/CH_2_Cl_2_ (1:1:1). Peptide condensation was achieved using HCl·L-Ala-D-*iso*-Gln(OBn), HATU, and triethylamine in DMF. The cleavage of the PGN fragments from JJ-Wang was performed with TFA/CH_2_Cl_2_/THF/H_2_O (5:4:1:0.2). Glycosylation yield was calculated by the absorbance of 9-methylidenefluorene after deprotection of Fmoc group with 30% Et_3_N in CH_2_Cl_2_. The compounds cleaved from JJ-Wang were isolated and the yields were calculated.

### 3.3. Purification of the PGN Fragments

Compound **20** was purified via silica gel column chromatography (toluene/EtOAc) and LH-20 column chromatography (CHCl_3_/MeOH). Compound **1** was purified via silica gel column chromatography (CHCl_3_/MeOH), LH-20 column chromatography (CHCl_3_/MeOH) and HPLC (see Supplementary Materials). Other compounds were purified via silica gel column chromatography.

### 3.4. Analysis of the PGN Fragments

Compound **1** was analyzed via nuclear magnetic resonance (NMR), high-resolution mass spectrometer (HRMS) and thin-layer chromatography (TLC). Other PGN fragments were analyzed via nuclear magnetic resonance (NMR), high-resolution mass spectrometer (HRMS), infrared spectroscopy (IR), specific rotation, thin-layer chromatography (TLC), and melting point measurement (see Supplementary Materials).

## 4. Conclusions

In this study, we successfully synthesized octasaccharides bearing dipeptide **1** using solid-phase oligosaccharide synthesis (SPOS). The JandaJel™ resin, which has high swelling capacity, was employed to expect high reactivity of glycosylation [15]. The relatively stable *N*-phenyl-trifluoroacetimidate donors were suitable for glycosylation on the resin. The addition of THF suppressed the Lewis acidity of TMSOTf, thereby preventing retro-glycosylation of *p*-oxybenzyl groups on the resin. Notably, the amide and ether of the glycan chain reduced the glycosylation reactivity. However, the use of fluorous solvent C_4_F_9_OEt addressed this limitation. C_4_F_9_OEt concentrated the donor **2** on the resin, enabling efficient synthesis of PGN octasaccharide **20** in 19% yield (10 steps). Subsequent hydrolysis and acetylation proceeded smoothly to afford the carboxylic acid **21**. Furthermore, multiple peptide condensations of **21** with dipeptides were achieved. Finally, the cleavage of **4** from the resin using TFA yielded the PGN octasaccharide with dipeptide **1** in 2.3% yield (15 steps). While glycopeptide and glycolipid syntheses often require complex purification due to their poor physicochemical properties, SPOS, which eliminates the need for purification during synthesis, holds strong potential as a powerful strategy for advancing such complex synthetic processes.

## Figures and Tables

**Table 1:** Investigation of the introduction of monosaccharides onto the resin.

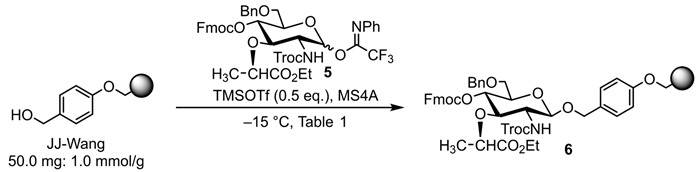				
Entry	Donor 5 (e.q.)	Solvent (5.0 *w*/*v* %)	Time (h)	Loading Ratio (%) *^1^
1 *^2^	1.0	CH_2_Cl_2_	1	22
2	2.0	CH_2_Cl_2_	1	35
3	3.0	CH_2_Cl_2_	1	45
4 *^2^	1.0	THF	1	34
5 *^2^	1.0	CH_2_Cl_2_/THF (9:1)	1	44
6	1.0	CH_2_Cl_2_/THF (9:1)	3	44

*^1^ Loading ratio was calculated by the absorbance of 9-methylidenefluorene after deprotection of Fmoc group with 30% Et_3_N in CH_2_Cl_2_. *^2^ Reference [38].

**Table 2:** Investigation of 2 + 2 glycosylation on the resin (**14**→**15**).

Entry	Solvent	Glycosylation Yield (%)
1	CH_2_Cl_2_/THF (9:1)	56
2	CH_2_Cl_2_/C_4_F_9_OEt/THF (5:4:1)	83

Glycosylation yield was calculated by the absorbance of 9-methylidenefluorene after deprotection of Fmoc group with 30% Et_3_N in CH_2_Cl_2_.

## Data Availability

All data are shown in the manuscript and the Supplementary Materials.

## Supplementary Materials

The following supporting information can be downloaded at: https://www.mdpi.com/article/10.3390/molecules30132787/s1, Scheme S1: Glycosylation of muramyl monosaccharide **5** onto JandaJel™ Wang resin; Scheme S2: Glycosylation of muramyl monosaccharide **8** onto Wang ChemMatrix^®^ resin; Scheme S3: Undesired deprotection and acetylation reaction during Ac capping; Scheme S4: Glycosylation of disaccharide **2** onto JJ-Wang and its cleavage from the resin; Scheme S5: Undesired cleavage of **10** with a Lewis acid; Scheme S6: Synthesis of octasaccharide **20**; Scheme S7: SPOS of the solid-supported tetrasaccharide **15**; Scheme S8: SPOS of octasaccharide **20**; Scheme S9: SPOS of octasaccharide with dipeptides **1**; Scheme S10: Synthesis of octasaccharide with dipeptides **S8**; Figure S1: MS-spectra of octasaccharide **20** (top: crude, bottom: pure); Table S1: HPLC conditions for the purification of **1**; Content 8: ^1^H- and ^13^C NMR spectra.
