# Supplementary material for: Solid-Phase Oligosaccharide Synthesis with Highly Complexed Peptidoglycan Fragments
Source: Molecules. 2025 Jun 28;30(13):2787. doi: 10.3390/molecules30132787 (PMC12251441; doi:10.3390/molecules30132787)

Supplementary Materials

Solid-phase Oligosaccharide Synthesis with Highly Complexed Peptidoglycan Fragments

Yuichiro Kadonaga,[1] Ning Wang,[2] Atsushi Shimoyama,[3] Yukari Fujimoto,*[4] Koichi Fukase*[1,3]

[1] Division of Science, Institute for Radiation Sciences, The University of Osaka, 1-1, Machikaneyama, Toyonaka, Osaka 560-0043, Japan; kadonagay19@irs.osaka-u.ac.jp (Y.K.); fukase.koichi.irs@osaka-u.ac.jp (K.F.)

[2] State Key Laboratory of Biopharmaceutical Preparation and Delivery, Institute of Process Engineering, Chinese Academy of Sciences, 1 North 2nd Street, Zhongguancun, Haidian District, Beijing 100190, China; wangning@mail.ipe.ac.cn (N.W.)

[3] Department of Chemistry, Graduate School of Science, The University of Osaka, 1-1, Machikaneyama, Toyonaka, Osaka 560-0043, Japan; ashimo@chem.sci.osaka-u.ac.jp (A.S.)

[4] Department of Chemistry, Faculty of Science and Technology, Keio University, 3-14-1 Hiyoshi, Kohoku-ku, Yokohama, Kanagawa 223-8522, Japan; fujimotoy@chem.keio.ac.jp (Y.F.)

* Correspondence: fukase.koichi.irs@osaka-u.ac.jp (K.F.); fujimotoy@chem.keio.ac.jp (Y.F.)

Contents

1. General method

^{1}H- and ^{13}C-NMR spectra were recorded on a JEOL ECA 500 (^{1}H, 500 MHz; ^{13}C, 126 MHz) or Bruker AVANCE 700 (^{1}H, 700 MHz; ^{13}C, 176 MHz) spectrometer. The chemical shifts in $CDCl_3$ are listed as δ-values with respect to tetramethylsilane (TMS) used as an internal standard. ESI–QTOF MS spectra were obtained on a Bruker micrOTOF-QII mass spectrometer. HRMS profiles were recorded using a Thermo Fisher Scientific ESI-LIT Orbitrap mass spectrometer. IR spectra were recorded using a JASCO FT/IR 6100 spectrometer. Absorbance spectra were obtained with a JASCO UV/VIS V-530 spectrophotometer. Shimadzu HPLC systems and Nacalai Tesque COSMOSIL columns were used for analysis and preparative separation. Janda-Jel™ Wang resin was purchased from Sigma–Aldrich. CH_2Cl_2, THF (Wako Pure Chemical Industries, Ltd.), and C_4F_9OEt (TCI Co., Ltd.) were utilized for glycosylation on a solid support after drying with MS3A molecular sieves. Silica gel column chromatography was performed using Kieselgel 60 (Merck, 0.040–0.063 mm) or silica gel 60 N (Kanto Chemical Co., spherical, neutral, 0.040–0.050 mm) at a medium pressure (2–4 kg/cm^2). All other reagents and solvents were purchased from commercial sources.

2. Abbreviation list

Ac: Acetyl
Ala: Alanine
Bn: Benzyl
DMAP: *N*,*N*-Dimethylaminopyridine
DMF: *N*,*N*-Dimethylformamide
ESI: Electrospray ionization
Fmoc: 9-Fluorenylmethyloxycarbonyl
GlcNAc: *N*-Acetyl glucosamine
Gln: Glutamine
HATU: 1-[bis(dimethylamino)methylene]-1*H*-1,2,3-triazolo[4,5-b]pyridinium 3-oxid hexa-fluorophosphate
HPLC: High-performance liquid chromatography
HRMS: High-resolution mass spectrometry
IR: Infrared
MurNAc: *N*-Acetylmuramic acid
MS: Mass spectrometry
MS4A: Molecular sieves, 4 Å
NMR: Nuclear magnetic resonance
QTOF: Quadrupole time-of-flight
SPOS: Solid-phase oligosaccharide synthesis
TEA: Triethylamine
TFA: Trifluoroacetic acid
THF: Tetrahydrofuran
TMS: Tetramethylsilane
TMSOTf: Trimethylsilyl trifluoromethanesulfonate
Troc: 2,2,2-Trichloroethoxycarbonyl
UV/VIS: ultraviolet–visible

3. Investigation of the glycosylation and Ac capping of saccharides and their cleavage from the resin

3-1. Glycosylation of muranyl monosaccharide **5** onto JandaJel™ Wang resin (JJ-Wang)

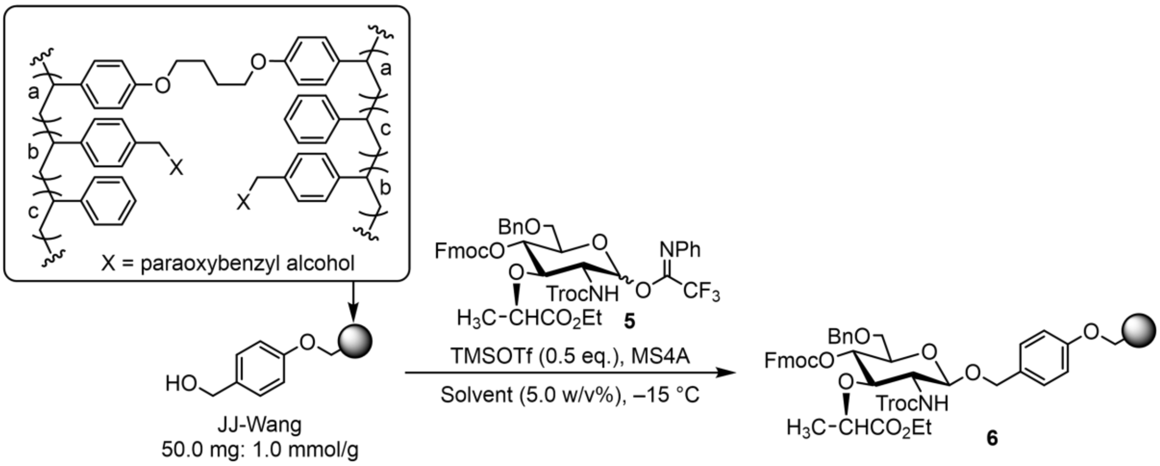

Scheme S1. Glycosylation of muramyl monosaccharide **5** onto JandaJel™ Wang resin.

TMSOTf (4.5 µL, 25 µmol) was added to a mixture of JJ-Wang resin (50.0 mg, 1.00 mmol/g), donor **5** (1.0–3.0 eq.), and MS4A (beads) in CH_2Cl_2 or CH_2Cl_2/THF (9:1) (1.0 mL) at −15 °C, and the reaction mixture was shaken at the same temperature for 1 or 3 h. After dilution and filtration with THF, the resin was washed and filtered with THF and CH_2Cl_2 (each 2 min, 3 times), CH_2Cl_2/MeOH (5:1), MeOH, CH_2Cl_2/MeOH (5:1) and CH_2Cl_2 (each 2 min, 2 times), and CH_2Cl_2 (2 min, 2 times). After washing and removing MS4A, the solid-supported monosaccharide **6** was dried *in vacuo*.

To calculate the loading yield, **6** was shaken with 30% Et_3N in CH_2Cl_2 at room temperature to deprotect Fmoc groups. After the reaction was complete, the produced 9-methylene-fluorene was collected, and absorbance was measured via UV–vis spectroscopy (λ_{max} = 301 nm, ε= 7800 $M \cdot cm^{-1}$).

3-2. Glycosylation of muranyl monosaccharide **5** onto ChemMatrix® resin (Wang CM)

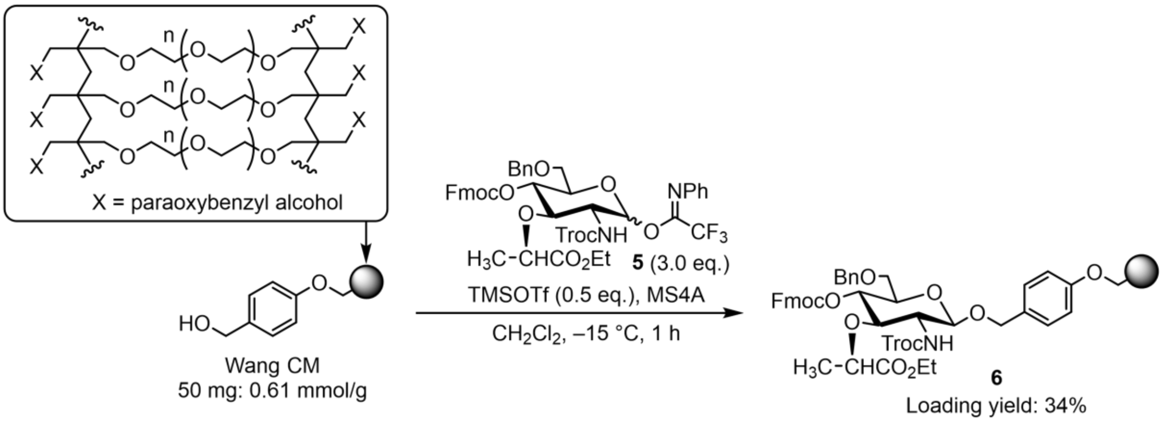

Scheme S2. Glycosylation of muramyl monosaccharide **8** onto Wang ChemMatrix® resin.

TMSOTf (2.80 µL, 15.3 µmol) was added to a mixture of Wang CM (50.0 mg, 0.61 mmol/g), donor **5** (85.8 mg, 91.5 µmol), and MS4A (beads) in CH_2Cl_2 (1.2 mL) at −15 °C, and the reaction mixture was shaken at the same temperature for 1 h. After dilution and filtration with THF, the resin was washed and filtered with THF and CH_2Cl_2 (each 2 min, 3 times), CH_2Cl_2/MeOH (5:1), MeOH, CH_2Cl_2/MeOH (5:1) and CH_2Cl_2 (each 2 min, 2 times), and CH_2Cl_2 (2 min, 2 times). After washing and removing MS4A, the solid-supported monosaccharide **6** was dried *in vacuo* overnight.

To calculate the loading yield, **6** was shaken with 30% Et_3N in CH_2Cl_2 at room temperature to deprotect Fmoc groups. After the reaction was complete, the produced 9-methylene-fluorene was collected, and absorbance was measured via UV–vis spectroscopy (λ_{max} = 301 nm, ε = 7800 M·cm^{-1}). The loading yield was 34%.

3-3. Ac capping of unreacted hydroxy groups on JandaJel™ Wang resin (JJ-Wang)

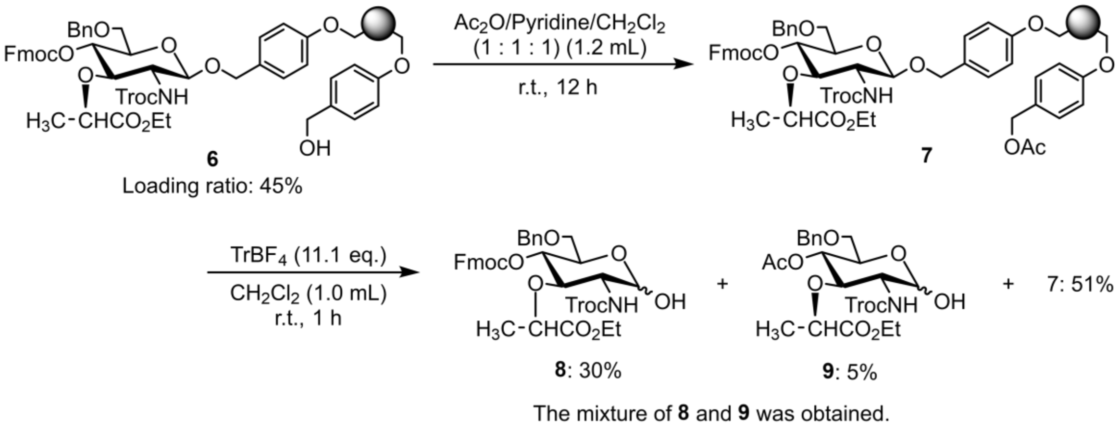

Scheme S3. Undesired deprotection and acetylation reaction during Ac capping.

A suspension of monosaccharides on the resin **6** (13.5 μmol) in Ac_2O/pyridine/CH_2Cl_2 (1:1:1) (1.2 mL) was shaken at 20 °C for 12 h. After dilution and filtration with CH_2Cl_2, the resin was washed and filtered with CH_2Cl_2 and THF (each 2 min, 4 times), CH_2Cl_2 and CH_2Cl_2/MeOH (5:1) (each 2 min, 4 times), and CH_2Cl_2 (2 min, 2 times). After washing, the solid-supported disaccharide **7** was dried *in vacuo*.

$TrBF_4$ (49.5 mg, 150 μmol) was added to a suspension of **7** (13.5 μmol) in CH_2Cl_2 (1.0 mL) at room temperature, and the reaction mixture was shaken at the same temperature for 1 h. After dilution and filtration with CH_2Cl_2, the resin was washed and filtered with CH_2Cl_2 (2 min, 3 times). The filtrate was neutralized with an aqueous $NaHCO_3$ solution at 0 °C. The organic layer was diluted with $CHCl_3$, washed with H_2O and brine, and dried over Na_2SO_4. After concentration and short silica gel column chromatography, a mixture of **8** (30%) and **9** (5%) was obtained.

To calculate the loading yield, compound **7** was shaken with 30% Et_3N in CH_2Cl_2 at room temperature to deprotect Fmoc groups. After the reaction was complete, the produced 9-methylene-fluorene was collected, and absorbance was measured via UV–vis spectroscopy (λ_{max} = 301 nm, ε= 7800 M·cm^{-1}). The remaining monosaccharide content was 51%.

3-4. SPOS of disaccharide **11**

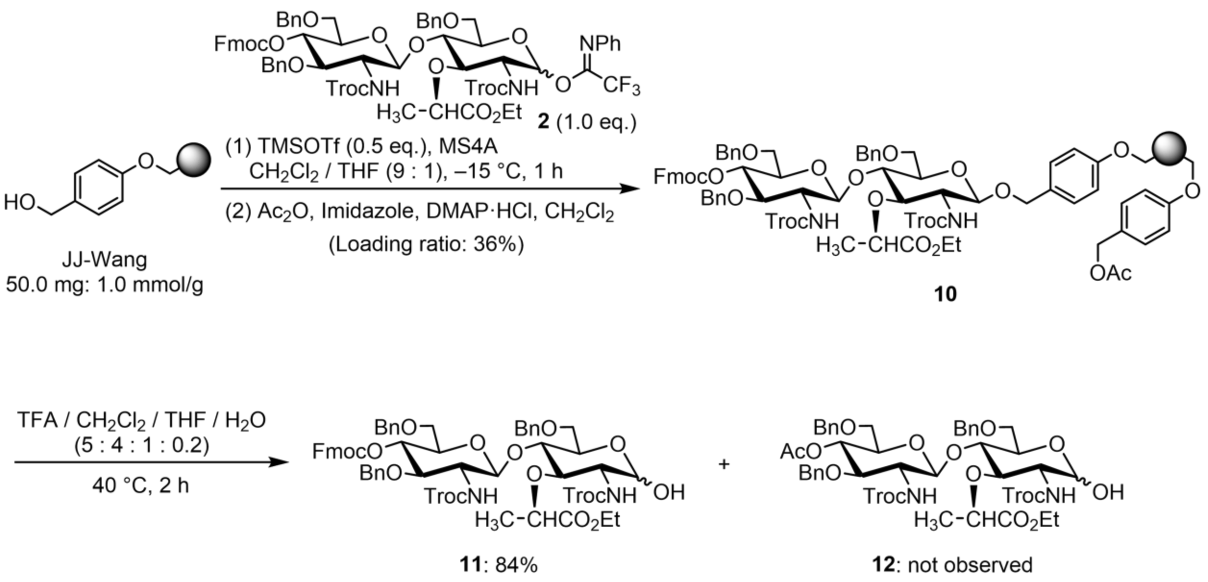

Scheme S4. Glycosylation of disaccharide **2** onto JJ-Wang and its cleavage from the resin.

Solid-supported disaccharide **10**

TMSOTf (5.4 μL, 30 μmol) was added to a mixture of JJ-Wang (60.0 mg, 1.00 mmol/g), donor **2** (87.3 mg, 60.0 μmol), and MS4A (beads) in CH_2Cl_2/THF (9:1) (1.2 mL) at −15 °C, and the reaction mixture was shaken at the same temperature for 1 h. After dilution and filtration with THF, the resin was washed and filtered with THF and CH_2Cl_2 (each 2 min, 3 times), CH_2Cl_2/MeOH (5:1), MeOH, CH_2Cl_2/MeOH (5:1) and CH_2Cl_2 (each 2 min, 2 times), and CH_2Cl_2 (2 min, 2 times). MS4A was removed during resin treatment for the next reaction. The loading ratio of the disaccharides was 36%.

Ac_2O (908 μL, 9.60 mmol), DMAP·HCl (47.6 mg, 300 μmol), and imidazole (81.7 mg, 1.20 mmol) were added to a suspension of disaccharide on the resin (the content of unreacted hydroxyl groups: 38.4 μmol) in CH_2Cl_2 (1.2 mL) at 20 °C, and the reaction mixture was shaken at the same temperature overnight. After dilution and filtration with CH_2Cl_2, the resin was washed and filtered with CH_2Cl_2 and THF (each 2 min, 4 times), CH_2Cl_2 and CH_2Cl_2/MeOH (5:1) (each 2 min, 4 times), and CH_2Cl_2 (2 min, 3 times). After washing, the solid-supported disaccharide **10** was dried *in vacuo*.

Disaccharide **11**

After the suspension of **10** (7.20 μmol) in CH_2Cl_2 was shaken for several minutes, the solvent was replaced with TFA/CH_2Cl_2/THF/H_2O (5:4:1:0.2), and the reaction mixture was stirred at 40 °C for 2 h. After dilution and filtration with CH_2Cl_2, the resin was washed and filtered with CH_2Cl_2 (2 min, 3 times). The filtrate was neutralized with an aqueous $NaHCO_3$ solution at 0 °C. The organic layer was diluted with $CHCl_3$, washed with H_2O and brine, and dried over Na_2SO_4. After concentration, silica gel column chromatography (toluene/EtOAc = 8:1, 5:1, 3:1, and 1:1) revealed the presence of alcohol **11** (7.8 mg, 84%), and the undesired product **12** was not detected.

3-5. Undesired cleavage of disaccharide **10** with a Lewis acid on JandaJel™ Wang resin (JJ-Wang)

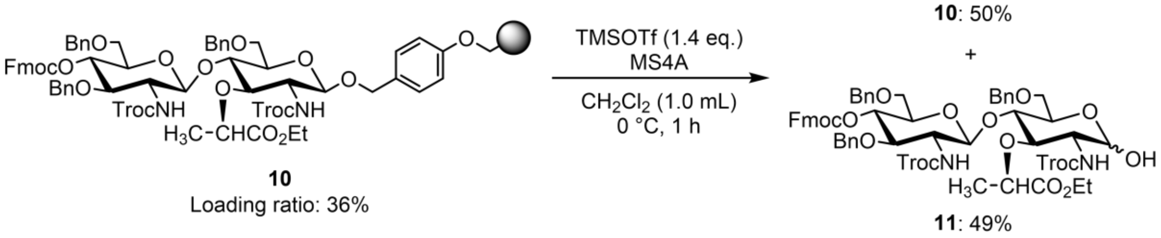

Scheme S5. Undesired cleavage of **10** with a Lewis acid.

TMSOTf (2.7 µL, 25 µmol) was added to a mixture of **10** (10.8 µmol) and MS4A (beads) in CH_2Cl_2 (1.0 mL) at 0 °C, and the reaction mixture was shaken at the same temperature for 1 h. After dilution and filtration with CH_2Cl_2, the resin was washed and filtered with CH_2Cl_2 (2 min, 3 times). The filtrate was neutralized with an aqueous $NaHCO_3$ solution at 0 °C. The organic layer was diluted with $CHCl_3$, washed with H_2O and brine, and dried over Na_2SO_4. After concentration, silica gel column chromatography (toluene/EtOAc = 8:1, 5:1, 3:1, 1:1) yielded disaccharide **11** (6.8 mg, 49%).

To calculate the remaining disaccharide content, compound **10** was shaken with 30% Et_3N in CH_2Cl_2 at room temperature to deprotect Fmoc groups. After the reaction was complete, the produced 9-methylene-fluorene was collected, and absorbance was measured via UV–vis spectroscopy (λ_{max} = 301 nm, ε= 7800 $M\cdot cm^{-1}$). The remaining disaccharide content was 50%.

4. Solution synthesis of octasaccharide 20

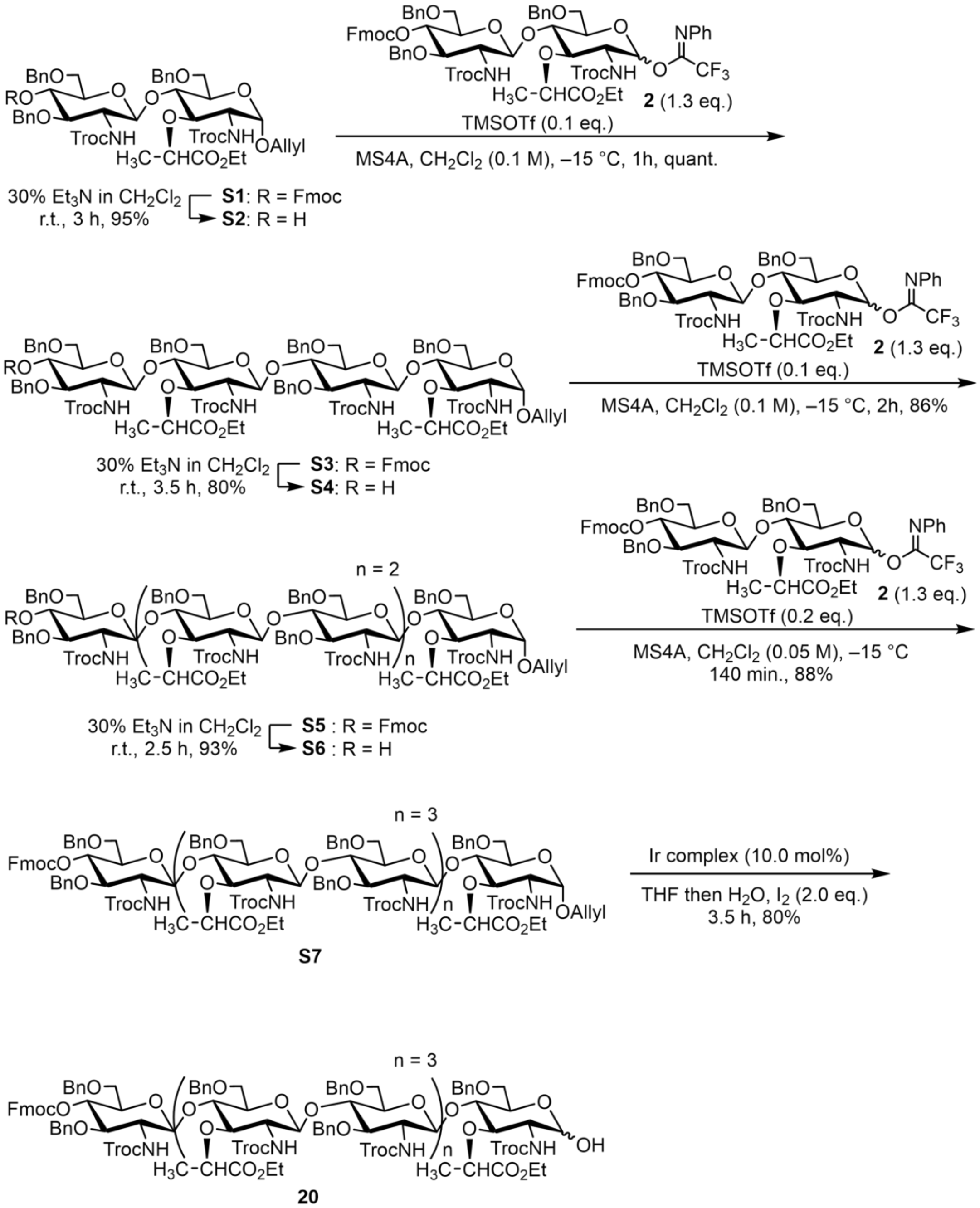

Scheme S6. Synthesis of octasaccharide **20**.

Disaccharide **S2**

A solution of the Fmoc-protected disaccharide **S1** (1.0 g, 0.76 mmol) with 30% Et_3N in CH_2Cl_2 (8.0 mL) was stirred at room temperature for 3 h. After co-evaporation with toluene, silica gel column chromatography (toluene/AcOEt, 10:1, 5:1, 3:1) produced disaccharide acceptor **S2** (0.79 g, 95%) as a white solid. R_f = 0.38 (toluene/AcOEt = 3:1); mp 55–59 °C; $[\alpha]^{25}_{D}$ +28.4 (*c* 0.51, $CHCl_3$); IR (neat) 3322, 2869, 1717 cm^{-1}; ^{1}H–NMR (500 MHz, $CDCl_3$) δ 7.42 (d, *J* = 4.4 Hz, 4H), 7.38 (brd, *J* = 2.0 Hz, 1H), 7.38–7.33 (m, 4H), 7.33–7.24 (m, 7H), 5.82 (ddt, *J* = 16.3, 11.3, 5.7 Hz, 1H), 5.24 (dd, *J* = 17.1, 1.5 Hz, 1H), 5.23–5.21 (m, 1H), 5.14 (dd, *J* = 10.5, 1.5 Hz, 1H), 4.87–4.75 (m, 1H), 4.84 (d, *J* = 12.1 Hz, 1H), 4.79 (d, *J* = 12.2 Hz, 1H), 4.79 (d, *J* = 11.6 Hz, 1H), 4.67 (d, *J* = 12.0 Hz, 1H), 4.66 (d, *J* = 11.6 Hz, 1H), 4.59 (*q*, *J* = 7.0 Hz, 1H), 4.62–4.56 (m, 1H), 4.56 (d, *J* = 11.7 Hz, 1H), 4.52 (d, *J* = 11.9 Hz, 1H), 4.32 (d, *J* = 11.9 Hz, 1H), 4.24 (d*q*, *J* = 10.8, 7.1 Hz, 1H), 4.20–4.15 (m, 1H), 4.13 (d*q*, *J* = 10.9, 7.2 Hz, 1H), 4.06 (ddt, *J* = 13.1, 5.3, 1.3 Hz, 1H), 3.95

(ddt, J = 13.2, 6.1, 1.2 Hz, 1H), 3.94–3.87 (m, 2H), 3.79 (dd, J = 9.8, 4.6 Hz, 1H), 3.76–3.66 (m, 3H), 3.66–3.61 (m, 1H), 3.65 (d, J = 4.5 Hz, 1H), 3.59 (d, J = 10.0 Hz, 1H), 3.44 (dd, J = 10.8, 1.9 Hz, 1H), 3.37 (brd, J = 7.9 Hz, 1H), 3.25 (dt, J = 9.7, 4.9 Hz, 1H), 3.07 (brs, 1H), 3.00 (s, 1H), 1.29 (d, J = 7.0 Hz, 3H), 1.27 (d, J = 7.2 Hz, 3H); ^{13}C NMR (126 MHz, $CDCl_3$) δ 175.4, 154.9, 154.0, 153.9, 138.4, 137.6, 137.3, 133.8, 129.7, 129.0, 128.6, 128.5, 128.0, 127.8, 127.7, 127.7, 117.4, 100.3, 96.7, 96.1, 96.0, 95.6, 81.6, 77.4, 75.0, 74.9, 74.5, 74.3, 74.2, 73.7, 73.7, 72.6, 70.9, 70.1, 68.9, 67.3, 61.4, 61.3, 57.0, 55.6, 55.4, 18.6, 14.1; HRMS (ESI-LIT Orbitrap) calcd. for $C_{47}H_{56}Cl_6N_2O_{15}Na$ $[M+Na]^+$ 1021.1704, found 1021.1703.

Tetrasaccharide **S3**

TMSOTf (9.9 μL, 55 μmol) was added to a mixture of acceptor **S2** (600.0 mg, 544.6 μmol), glucosaminyl donor **2** (1.03 g, 708 mmol), and MS4A (600 mg) in CH_2Cl_2 (5.4 mL), which was subsequently stirred at −15 °C for 1 h. After the reaction was quenched with EtOH and aqueous $NaHCO_3$, the mixture was diluted with EtOAc. The organic layer was washed with H_2O and brine and then dried over Na_2SO_4. After concentration, silica gel column chromatography (toluene/EtOAc, 10:1, 8:1, and 6:1) resulted in tetrasaccharide **S3** (1.29 g, quant.) as a white solid. R_f = 0.48 (toluene/AcOEt = 5:1); mp 78–86 °C; $[\alpha]^{25}_D$ +3.5 (c 0.56, $CHCl_3$); IR (neat) 2868, 1719 cm^{-1}; ^{1}H–NMR (500 MHz, $CDCl_3$) δ 7.76 (d, J = 6.9 Hz, 1H), 7.74 (d, J = 6.7 Hz, 1H), 7.57 (d, J = 7.5 Hz, 1H), 7.52 (d, J = 7.6 Hz, 1H), 7.48–7.19 (m, 33H), 7.44 (d, J = 7.2 Hz, 1H), 7.17 (d, J = 7.7 Hz, 1H), 7.17 (d, J = 6.7 Hz, 1H), 7.05 (s, 1H), 5.82 (ddt, J = 16.8, 10.9, 5.6 Hz, 1H), 5.27–5.23 (m, 1H), 5.23 (dd, J = 17.2, 1.5 Hz, 1H), 5.13 (dd, J = 10.5, 1.3 Hz, 1H), 5.10 (d, J = 12.4 Hz, 1H), 4.90–4.74 (m, 4 H), 4.85 (t, J = 9.4 Hz, 1H), 4.80 (d, J = 12.2 Hz, 1H), 4.73–4.60 (m, 4H), 4.68 (d, J = 12.0 Hz, 1H), 4.60–4.52 (m, 1H), 4.56 (d, J = 13.2 Hz, 1H), 4.54 (d, J = 11.7 Hz, 1H), 4.51–4.36 (m, 3 H), 4.46 (d, J = 11.3 Hz, 1H), 4.46 (s, 2H), 4.39 (d, J = 12.1 Hz, 1H), 4.36–4.19 (m, 4H), 4.34 (t, J = 7.3 Hz, 1H), 4.33 (d, J = 7.5 Hz, 1H), 4.31 (d, J = 6.8 Hz, 1H), 4.18–4.01 (m, 2H), 4.15 (t, J = 7.3 Hz, 1H), 4.12 (d, J = 6.9 Hz, 1H), 4.10 (d, J = 7.1 Hz, 1H), 4.05 (dd, J = 13.0, 5.3 Hz, 1H), 4.00–3.83 (m, 4H), 3.95 (d, J = 13.0, 6.0 Hz, 1H), 3.90 (d, J = 9.3 Hz, 1H), 3.74 (d, J = 9.6 Hz, 1H), 3.72 (d, J = 10.2 Hz, 1H), 3.67–3.54 (m, 3H), 3.64 (d, J = 6.0 Hz, 1H), 3.64 (dd, J = 10.7, 4.0 Hz, 1H), 3.58 (d, J = 10.3, 5.1 Hz, 1H), 3.51–3.31 (m, 4H), 3.48 (d, J = 9.5 Hz, 1H), 3.35 (d, J = 8.9 Hz, 1H), 3.15 (t, J = 9.5 Hz, 1H), 3.10 (d, J = 7.5 Hz, 1H), 3.01 (brs, 1H), 2.85 (d, J = 8.2 Hz, 1H), 1.35 (d, J = 6.8 Hz, 3H), 1.30 (d, J = 7.0 Hz, 3H), 1.29 (t, J = 7.2 Hz, 3H), 1.27 (t, J = 7.2 Hz, 3H); ^{13}C NMR (126 MHz, $CDCl_3$) δ 175.7, 175.5, 156.0, 154.9, 154.2, 153.9, 153.8, 143.3, 143.1, 141.3, 141.3, 139.4, 137.9, 137.7, 137.6, 137.5, 133.8, 129.8, 129.2, 129.0, 128.4, 128.4, 128.3, 128.1, 127.9, 127.9, 127.8, 127.7, 127.6, 127.5, 127.3, 127.1, 125.0, 124.9, 120.1, 117.4, 102.1, 100.7, 99.8, 96.2, 96.1, 96.0, 95.7, 95.4, 80.7, 79.3, 77.5, 77.3, 76.0, 75.2, 75.0, 74.6, 74.6, 74.5, 74.4, 74.3, 74.2, 74.2, 74.0, 73.8, 73.8, 73.5, 73.4, 72.9, 72.4, 70.2, 70.0, 69.4, 68.9, 68.9, 68.3, 67.9, 67.0, 61.3, 61.2, 57.5, 57.0, 55.3, 46.7, 18.5, 18.4, 14.1, 14.1; HRMS (ESI-LIT Orbitrap) calcd. for $C_{106}H_{116}Cl_{12}N_4O_{31}Na$ $[M+Na]^+$ 2383.3778, found 2383.3779.

Tetrasaccharide **S4**

A solution of the Fmoc-protected tetrasaccharide **S3** (400.0 mg, 169.0 μmol) with 30% Et_3N in CH_2Cl_2 (2.0 mL) was stirred at room temperature for 3.5 h. After co-evaporation with toluene, silica gel column chromatography (toluene/EtOAc, 10:1, 5:1, 3:1) produced tetrasaccharide acceptor **S4** (289.9 mg, 80%) as a white solid. R_f = 0.44: (toluene/AcOEt = 3:1); mp 82–86 °C; $[\alpha]^{25}_D$ +2.3 (c 0.51, $CHCl_3$); IR (neat) 3392, 3307, 2870, 1718 cm^{-1}; ^{1}H–NMR (500 MHz, $CDCl_3$) δ 7.48–7.41 (m, 3H), 7.40–7.35 (m, 6H), 7.35–7.20 (m, 22H), 7.02 (s, 1H), 5.82 (ddt, J = 16.8, 11.1, 5.6 Hz, 1H), 5.27–5.20 (m, 1H), 5.24 (s, 1H), 5.13 (dd, J = 10.5, 1.4 Hz, 1H), 5.10 (d, J = 13.5 Hz, 1H), 4.88–4.35 (m, 11H), 4.85 (d, J = 12.0 Hz, 1H), 4.84 (d, J = 12.2 Hz, 1H), 4.80 (d, J = 12.1 Hz, 1H), 4.73 (d, J = 11.6 Hz, 1H), 4.67 (d, J = 12.2 Hz, 1H), 4.62 (dd, J = 9.2, 2.0 Hz, 1H), 4.56 (d, J = 11.9 Hz, 1H), 4.54 (d, J = 8.6 Hz, 1H), 4.38 (d, J = 11.7

Hz, 1H), 4.31–4.17 (m, 3H), 4.27 (dd, J = 10.7, 7.1 Hz, 1H), 4.20 (d, J = 8.3 Hz, 1H), 4.17–4.10 (m, 1H), 4.13 (dd, J = 10.8, 7.1 Hz, 1H), 4.05 (dd, J = 13.0, 5.3 Hz, 1H), 4.01 (d, J = 12.5 Hz, 1H), 4.08–3.92 (m, 4H), 3.92–3.81 (m, 2H), 3.87 (t, J = 9.0 Hz, 2H), 3.79–3.06 (m, 3H), 3.76 (dd, J = 9.8, 4.8 Hz, 1H), 3.69 (dd, J = 9.8, 5.3 Hz, 1H), 3.60–3.54 (m, 2H), 3.50 (d, J = 8.9 Hz, 1H), 3.42 (d, J = 9.8 Hz, 2H), 3.35 (t, J = 10.1 Hz, 2H), 3.22 (dt, J = 9.7, 4.9 Hz, 1H), 3.17–3.06 (m, 2H), 3.06–2.96 (m, 2H), 2.93 (d, J = 1.7 Hz, 1H), 2.85 (d, J = 8.6 Hz, 1H), 1.35 (d, J = 6.9 Hz, 3H), 1.28 (t, J = 6.6 Hz, 3H), 1.28 (d, J = 6.2 Hz, 3H), 1.27 (t, J = 6.5 Hz, 3H); ^{13}C NMR (126 MHz, $CDCl_3$) δ 175.7, 175.5, 156.0, 154.9, 154.0, 153.9, 139.4, 139.4, 138.3, 137.8, 137.5, 137.5, 137.3, 133.8, 130.0, 129.3, 129.2, 129.0, 128.6, 128.5, 128.4, 128.1, 128.0, 127.9, 127.8, 127.7, 127.7, 127.3, 127.1, 117.4, 102.0, 100.6, 100.1, 96.2, 96.1, 96.0, 95.7, 95.7, 95.5, 81.8, 80.7, 75.2, 75.0, 74.6, 74.5, 74.4, 74.2, 73.8, 73.7, 73.7, 73.6, 73.5, 72.9, 70.9, 70.2, 68.9, 68.3, 68.0, 67.0, 67.0, 61.3, 61.2, 57.1, 57.0, 55.3, 18.5, 18.4, 14.1, 14.1; HRMS (ESI-LIT Orbitrap) calcd. for $C_{91}H_{106}Cl_{12}N_4O_{29}Na$ $[M+Na]^+$ 2161.3097, found 2161.3098.

Hexasaccharide **S5**

TMSOTf (4.0 μL, 22 μmol) was added to a mixture of acceptor **S4** (477.0 g, 222.4 μmol), glucosaminyl donor **2** (420.6 mg, 289.1 mmol), and MS4A (477 mg) in CH_2Cl_2 (2.2 mL), and the mixture was stirred at −15 °C for 2 h. After the reaction was quenched with MeOH and aqueous $NaHCO_3$ solution, the mixture was diluted with EtOAc. The organic layer was washed with H_2O and brine and then dried over Na_2SO_4. After concentration, silica gel column chromatography (toluene/EtOAc, 8:1, 6:1, and 4:1) produced hexasaccharide **S5** (665.7 mg, 86%) as a white solid. R_f = 0.35 (toluene/AcOEt = 5:1); mp 85–93 °C; $[\alpha]^{25}_D$ −6.4 (c 0.54, $CHCl_3$); IR (neat) 3320, 2867, 1719 cm^{-1}; ^{1}H–NMR (500 MHz, $CDCl_3$) δ 7.76 (d, J = 7.0 Hz, 1H), 7.75 (d, J = 6.8 Hz, 1H), 7.57 (dd, J = 7.5, 0.5 Hz, 1H), 7.52 (dd, J = 7.5, 0.5 Hz, 1H), 7.47–7.42 (m, 2H), 7.42–7.35 (m, 9H), 7.34–7.19 (m, 38H), 7.19–7.14 (m, 2H), 7.05 (s, 2H), 5.82 (ddt, J = 16.0, 11.1, 5.6 Hz, 1H), 5.28–5.23 (m, 1H), 5.23 (dd, J = 17.2, 1.6 Hz, 1H), 5.13 (dd, J = 10.4, 1.4 Hz, 1H), 5.11 (d, J = 11.6 Hz, 1H), 5.07 (d, J = 12.3 Hz, 1H), 4.89–4.73 (m, 4H), 4.80 (d, J = 11.9 Hz, 2H), 4.78 (t, J = 12.5 Hz, 2H), 4.70–4.58 (m, 3H), 4.67 (d, J = 12.5 Hz, 2H), 4.63 (dd, J = 6.9, 3.2 Hz, 2H), 4.60–4.51 (m, 2H), 4.55 (d, J = 11.6 Hz, 2H), 4.54 (d, J = 11.2 Hz, 2H), 4.50–4.35 (m, 3H), 4.46 (d, J = 5.0 Hz, 2H), 4.45 (d, J = 6.3 Hz, 2H), 4.42 (brd, J = 5.3 Hz, 1H), 4.35–4.22 (m, 4H), 4.33 (d, J = 9.5 Hz, 1H), 4.31 (d, J = 9.0 Hz, 1H), 4.25 (d, J = 6.7 Hz, 1H), 4.22–4.08 (m, 3H), 4.19 (t, J = 8.3 Hz, 1H), 4.15 (t, J = 7.2 Hz, 1H), 4.13 (d, J = 7.7 Hz, 1H), 4.10 (d, J = 7.2 Hz, 1H), 4.08–3.78 (m, 12H), 4.05 (dd, J = 13.1, 5.4 Hz, 2H), 3.75–7.68 (m, 3H), 3.67–3.53 (m, 5H), 3.63 (dd, J = 1.05, 4.0 Hz, 2H), 3.50–3.46 (m, 2H), 3.46–3.37 (m, 4H), 3.37–3.29 (m, 3H), 3.21–2.94 (m, 5H), 3.15 (q, J = 10.0 Hz, 1H), 2.87 (d, J = 9.0 Hz, 1H), 2.83 (d, J = 7.2 Hz, 1H), 1.38–1.23 (m, 9H), 1.34 (d, J = 6.8 Hz, 3H), 1.29 (t, J = 7.2 Hz, 3H), 1.29 (d, J = 7.0 Hz, 3H); ^{13}C NMR (126 MHz, $CDCl_3$) δ 175.7, 175.7, 175.5, 156.0, 154.9, 154.2, 154.0, 153.8, 143.3, 143.1, 141.3, 141.3, 139.4, 139.3, 137.9, 137.8, 137.6, 137.6, 137.5, 133.8, 129.4, 129.2, 129.1, 129.0, 128.5, 128.5, 128.4, 128.3, 128.1, 127.9, 127.9, 127.8, 127.7, 127.6, 127.5, 127.4, 127.3, 127.2, 127.1, 125.0, 124.9, 120.1, 117.4, 96.2, 96.0, 95.5, 95.4, 80.7, 77.5, 77.3, 76.0, 75.2, 75.0, 74.8, 74.6, 74.6, 74.5, 74.4, 74.4, 74.3, 74.2, 74.1, 74.0, 73.8, 73.7, 73.5, 73.4, 72.9, 72.4, 70.2, 70.0, 69.4, 68.9, 68.3, 67.9, 67.4, 67.0, 61.3, 61.2, 57.5, 57.0, 55.4, 46.7, 29.7, 18.5, 18.4, 18.4, 14.1, 14.1, 14.1; HRMS (ESI-LIT Orbitrap) calcd. for $C_{150}H_{166}Cl_{18}N_6O_{45}Na_2$ $[M+2Na]^{2+}$ 1723.2532, found 1723.2555.

Hexasaccharide **S6**

A solution of the Fmoc-protected hexasaccharide **S5** (200 mg, 58.6 μmol) with 30% Et_3N in CH_2Cl_2 (1.0 mL) was stirred at room temperature for 2.5 h. After co-evaporation with toluene, silica gel column chromatography (toluene/EtOAc, 8:1, 5:1, 3:1) produced hexasaccharide acceptor **S6** (173.2 mg, 93%) as a white solid. R_f = 0.45 (toluene/AcOEt = 3:1);

mp 88–93 °C; $[\alpha]^{25}_{D}$ –8.7 (*c* 0.57, $CHCl_3$); IR (neat) 3320, 2868, 1718 cm^{-1}; ^{1}H–NMR (500 MHz, $CDCl_3$) δ 7.49–7.41 (m, 3H), 7.42–7.34 (m, 10H), 7.34–7.19 (m, 33H), 7.06 (s, 1H), 7.01 (s, 1H), 5.81 (ddt, *J* = 16.7, 11.1, 5.5 Hz, 1H), 5.27–5.22 (m, 1H), 5.23 (dd, *J* = 16.9, 1.6 Hz, 1H), 5.12 (dd, *J* = 10.4, 1.4 Hz, 1H), 5.10 (d, *J* = 11.8 Hz, 1H), 5.07 (d, *J* = 12.9 Hz, 1H), 4.88–4.70 (m, 3H), 4.85 (dd, *J* = 12.2, 1.0 Hz, 1H), 4.84 (d, *J* = 10.9 Hz, 1H), 4.82 (d, *J* = 7.2 Hz, 1H), 4.78 (d, *J* = 9.9 Hz, 1H), 4.73 (d, *J* = 11.6 Hz, 1H), 4.70–4.49 (m, 13H), 4.67 (d, *J* = 12.5 Hz, 1H), 4.62 (d, *J* = 11.5 Hz, 1H), 4.49–4.34 (m, 3H), 4.38 (d, *J* = 12.5 Hz, 2H), 4.31–4.09 (m, 6H), 4.27 (td, *J* = 6.9, 4.0 Hz, 1H), 4.23 (td, *J* = 7.5, 3.4 Hz, 1H), 4.20 (brs, 1H), 4.18 (brs, 1H), 4.08–3.91 (m, 7H), 4.05 (dd, *J* = 13.1, 5.3 Hz, 2H), 3.91–3.79 (m, 5H), 3.87 (d, *J* = 9.2 Hz, 1H), 3.79–3.61 (m, 7H), 3.76 (dd, *J* = 9.8, 4.7 Hz, 1H), 3.61–3.53 (m, 3H), 3.49 (t, *J* = 9.2 Hz, 2H), 3.42 (d, *J* = 9.3 Hz, 2H), 3.53–3.29 (m, 4H), 3.22 (dt, *J* = 9.6, 4.8 Hz, 1H), 3.18–2.96 (m, 7H), 2.90 (s, 1H), 2.92–2.78 (m, 2H), 1.34 (d, *J* = 6.9 Hz, 3H), 1.32 (d, *J* = 7.2 Hz, 3H), 1.29 (t, *J* = 7.3 Hz, 3H), 1.28 (t, *J* = 7.1 Hz, 3H), 1.31–1.24 (m, 6H); ^{13}C NMR (126 MHz, $CDCl_3$) δ 175.7, 175.7, 175.5, 156.0, 154.9, 154.0, 139.4, 139.3, 138.3, 137.9, 137.8, 137.8, 137.5, 137.5, 137.5, 137.3, 133.8, 129.8, 129.3, 129.2, 129.1, 129.0, 128.6, 128.5, 128.4, 128.1, 128.0, 127.9, 127.9, 127.8, 127.7, 127.7, 127.4, 127.3, 127.1, 117.4, 102.1, 100.7, 100.3, 100.1, 96.2, 96.2, 96.1, 96.1, 96.0, 95.7, 95.5, 81.8, 81.8, 80.7, 75.2, 75.0, 74.8, 74.6, 74.5, 74.4, 74.3, 74.2, 74.0, 73.8, 73.7, 73.6, 73.5, 73.4, 72.9, 72.9, 70.9, 70.2, 68.9, 68.3, 67.9, 67.4, 67.0, 61.3, 61.2, 57.1, 57.0, 55.3, 18.5, 18.4, 18.4, 14.1, 14.1, 14.1; HRMS (ESI-LIT Orbitrap) calcd. for $C_{135}H_{156}Cl_{18}N_6O_{43}Na_2$ $[M+2Na]^{2+}$ 1612.2191, found 1612.2227.

Octasaccharide **S7**

TMSOTf (2.7 μL, 15 μmol) was added to a mixture of acceptor **S6** (240 mg, 75.3 μmol), glucosaminyl donor **2** (142 mg, 97.9 μmol), and MS4A (240 mg) in CH_2Cl_2 (1.5 mL), and the mixture was stirred at −15 °C for 2.5 h. After the reaction was quenched with EtOH and saturated aqueous $NaHCO_3$, the mixture was diluted with AcOEt. The organic layer was washed with H_2O and brine and then dried over Na_2SO_4. After concentration, silica gel column chromatography (toluene/EtOAc, 8:1, 6:1, and 4:1) produced octasaccharide **S7** (293.8 mg, 88%) as a white solid. R_f = 0.27 (toluene/AcOEt = 5:1); mp 95–99 °C; $[\alpha]^{25}_{D}$ −14.1 (*c* 0.50, $CHCl_3$); IR (neat) 3313, 2869, 1720 cm^{-1}; ^{1}H–NMR (500 MHz, $CDCl_3$) δ 7.76 (d, *J* = 6.9 Hz, 1H), 7.75 (d, *J* = 6.9 Hz, 1H), 7.57 (d, *J* = 7.5 Hz, 1H), 7.53 (d, *J* = 7.6 Hz, 1H), 7.48–7.42 (m, 2H), 7.45 (d, *J* = 6.9 Hz, 1H), 7.42–7.35 (m, 10H), 7.35–7.18 (m, 50H), 7.17 (d, *J* = 7.7 Hz, 1H), 7.17 (d, *J* = 7.0 Hz, 1H), 7.07 (brs, 2H), 5.82 (ddt, *J* = 16.7, 11.1, 5.5 Hz, 1H), 5.27–5.23 (m, 1H), 5.23 (dd, *J* = 17.3, 1.5 Hz, 1H), 5.16–5.04 (m, 3H), 5.13 (dd, *J* = 10.3, 1.2 Hz, 1H), 4.89–4.72 (m, 8H), 4.86 (d, *J* = 11.8 Hz, 1H), 4.80 (d, *J* = 11.9 Hz, 1H), 4.71–4.59 (m, 8H), 4.67 (d, *J* = 12.3 Hz, 1H), 4.60–4.50 (m, 7H), 4.54 (d, *J* = 11.8 Hz, 1H), 4.54 (d, *J* = 11.2 Hz, 1H), 4.49–4.35 (m, 7H), 4.45 (s, 2H), 4.45 (d, *J* = 11.3 Hz, 1H), 4.40 (d, *J* = 12.1 Hz, 1H), 4.35–4.09 (m, 14H), 4.33 (d, *J* = 9.6 Hz, 1H), 4.31 (d, *J* = 9.0 Hz, 1H), 4.24 (d, *J* = 7.3 Hz, 1H), 4.12 (*q*, *J* = 7.1 Hz, 1H), 4.09–3.77 (m, 18H), 4.05 (dd, *J* = 13.1, 5.4 Hz, 1H), 3.95 (dd, *J* = 12.0, 6.6 Hz, 1H), 3.77–3.67 (m, 4H), 3.67–3.52 (m, 8H), 3.63 (dd, *J* = 10.3, 3.7 Hz, 1H), 3.51–3.45 (m, 3H), 3.45–3.37 (m, 6H), 3.37–3.29 (m, 3H), 3.22–2.95 (m, 9H), 2.92–2.77 (m, 3H), 1.37–1.23 (m, 15H), 1.34 (d, *J* = 6.6 Hz, 3H), 1.29 (t, *J* = 7.1 Hz, 3H), 1.27 (t, *J* = 7.2 Hz, 3H); ^{13}C NMR (126 MHz, $CDCl_3$) δ 175.8, 175.7, 175.7, 175.5, 156.0, 154.9, 154.2, 154.0, 154.0, 153.8, 143.3, 143.1, 141.3, 141.3, 139.4, 139.3, 137.9, 137.8, 137.8, 137.6, 137.6, 137.5, 133.8, 129.8, 129.4, 129.2, 129.1, 129.1, 129.0, 128.5, 128.5, 128.4, 128.3, 128.1, 127.9, 127.9, 127.8, 127.7, 127.6, 127.5, 127.4, 127.3, 127.1, 127.1, 125.0, 124.9, 120.1, 117.4, 102.1, 100.7, 100.4, 99.8, 96.2, 96.2, 96.0, 95.5, 95.4, 95.4, 80.8, 79.3, 77.5, 77.3, 76.0, 75.2, 75.0, 74.7, 74.6, 74.6, 74.5, 74.4, 74.3, 74.2, 74.1, 73.8, 73.8, 73.7, 73.5, 73.4, 73.4, 72.9, 72.9, 72.4, 70.2, 70.0, 69.4, 68.9, 68.3, 67.9, 67.3, 67.0, 61.3, 61.2, 57.5, 57.0, 55.3, 46.7, 29.7, 18.5, 18.4, 18.4, 14.1, 14.1, 14.1; HRMS (ESI-LIT Orbitrap) calcd. for $C_{194}H_{216}Cl_{24}N_8O_{59}Na_3$ $[M+3Na]^{3+}$ 1503.2116, found 1503.2057.

Octasaccharide **20**

$[Ir(cod)(PMePh_2)_2]PF_6$ (1.7 mg, 2.0 μmol) was suspended in THF under Ar. After the activation of Ir complex with H_2, H_2 was replaced with Ar. A solution of **S7** (177.4 mg, 39.8 μmol) in THF (800 μL) was added to a solution of the activated Ir complex in THF (200 μL), and the reaction mixture was stirred at room temperature for 2.5 h. After the addition of the activated Ir complex (1.7 mg, 2.0 μmol) in THF (200 μL), the reaction mixture was stirred at the same temperature for 40 min. H_2O (330 μL) and I_2 (20.2 mg, 79.6 μmol) were added to the resulting mixture, which was then stirred for 20 min. After the reaction was quenched with a 10% aqueous $Na_2S_2O_3$ solution, the mixture was diluted with EtOAc. The organic layer was washed with aqueous $NaHCO_3$, H_2O, and brine and then dried over Na_2SO_4. After concentration, silica gel column chromatography (toluene/EtOAc = 5:1, 3:1, and 1:1) produced alcohol **20** (139.8 mg, 80%) as a white solid. R_f = 0.45 (toluene/EtOAc = 3:1); mp 99–105 °C; $[\alpha]^{25}_D$ −16.5 (*c* 0.51, $CHCl_3$); IR (neat) 3305, 2870, 1719 cm^{-1}; ^1H NMR (700 MHz, $CDCl_3$) δ 7.76 (d, *J* = 5.7 Hz, 1H), 7.75 (d, *J* = 5.7 Hz, 1H), 7.68 (s, 1H), 7.57 (d, *J* = 5.4 Hz, 1H), 7.52 (d, *J* = 5.4 Hz, 1H), 7.44 (t, *J* = 5.1 Hz, 2H), 7.42–7.35 (m, 10 H), 7.35–7.19 (m, 50H), 7.18–7.15 (m, 2H), 7.11–7.01 (m, 3H), 5.64 (s, 1H), 5.10 (d, *J* = 9.2 Hz, 1H), 5.10–5.04 (m, 2H), 4.89–4.79 (m, 4H), 4.85 (t, *J* = 7.0 Hz, 1H), 4.82 (d, *J* = 9.0 Hz, 1H), 4.79–4.70 (m, 4H), 4.70–4.60 (m, 7H), 4.62 (*q*, *J* = 5.4 Hz, 1H), 4.60–4.50 (m, 8H), 4.48–4.36 (m, 8H), 4.45 (d, *J* = 8.1 Hz, 1H), 4.45 (s, 2H), 4.45 (s, 2H), 4.36–4.21 (m, 7H), 4.32 (dd, *J* = 7.5, 5.3 Hz, 1H), 4.30 (dd, *J* = 7.6, 4.9 Hz, 1H), 4.22–4.10 (m, 6H), 4.19 (*q*, *J* = 5.8 Hz, 1H), 4.12 (*q*, *J* = 5.7 Hz, 1H), 4.10–3.77 (m, 18H), 3.90 (t, *J* = 6.7 Hz, 1H), 3.75–3.67 (m, 4H), 3.66–3.61 (m, 3H), 3.61–3.54 (m, 4H), 3.51–3.45 (m, 3H), 3.45–3.37 (m, 5H), 3.37–3.28 (m, 4H), 3.20–2.94 (m, 9H), 2.90–2.78 (m, 3H), 2.62 (d, *J* = 1.9 Hz, 1H), 1.35 (d, *J* = 4.1 Hz, 3H), 1.33–1.24 (m, 18H), 1.27 (d, *J* = 5.3 Hz, 3H); ^{13}C NMR (176 MHz, $CDCl_3$) δ 176.0, 175.8, 175.8, 175.6, 156.0, 155.1, 154.2, 154.0, 153.8, 143.3, 143.1, 141.3, 141.3, 139.4, 137.9, 137.8, 137.7, 137.6, 137.5, 129.7, 129.4, 129.3, 129.1, 129.0, 128.6, 128.5, 128.5, 128.4, 128.3, 128.2, 128.0, 127.9, 127.8, 127.7, 127.6, 127.5, 127.5, 127.4, 127.3, 127.2, 125.0, 125.0, 120.1, 102.1, 100.7, 100.4, 99.9, 96.2, 95.8, 95.6, 95.4, 90.9, 80.8, 80.7, 79.3, 77.5, 76.0, 75.3, 74.9, 74.8, 74.6, 74.6, 74.5, 74.5, 74.4, 74.3, 74.1, 73.8, 73.5, 73.4, 72.9, 72.4, 70.2, 70.0, 69.5, 68.3, 67.9, 67.3, 67.1, 61.3, 61.3, 61.2, 57.5, 57.0, 55.8, 46.7, 29.7, 18.5, 18.4, 14.1, 14.1, 14.1; HRMS (ESI-LIT Orbitrap) calcd. for $C_{191}H_{212}Cl_{24}N_8O_{59}Na_3$ $[M+3Na]^{3+}$ 1489.8679, found 1489.8620.

5. SPOS of octasaccharide 20

5-1. SPOS of tetrasacharide **15**

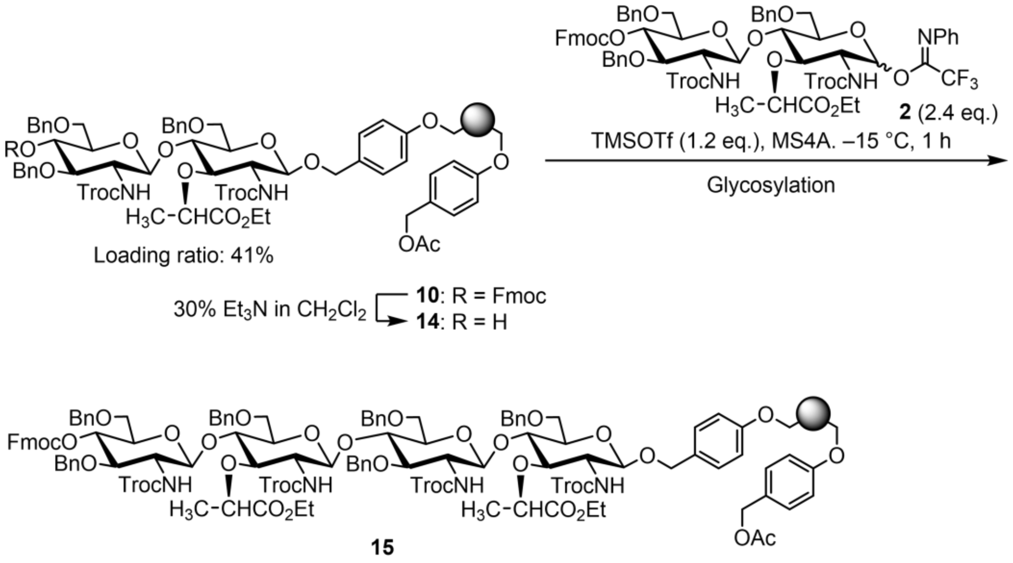

Glycosylation

Entry	Solvent	Glycosylation yield*
1	CH_2Cl_2/THF (9 : 1)	56%
2	CH_2Cl_2/C_4F_9OEt/THF (5 : 4 : 1)	83%

*Glycosylation yield was calculated by absorbance of 9-methylidenefluorene after depritection of Fmoc group with 30% Et_3N in CH_2Cl_2.

Scheme S7. SPOS of the solid-supported tetrasaccharide **15**.

Glycosyl acceptor **14**

After the suspension of **10** (28.7 μmol) in CH_2Cl_2 was shaken for several minutes, the solvent was replaced with 30% Et_3N in CH_2Cl_2 (3.5 mL) at 20 °C, and the resulting mixture was shaken at the same temperature for 6 h. After dilution and filtration with CH_2Cl_2, the resin was washed and filtered with CH_2Cl_2 and THF (each 2 min, 10 times) and then CH_2Cl_2 (2 min, 3 times). After washing, disaccharide acceptor **14** was dried *in vacuo*.

Solid-supported tetrasaccharide **15**

(Conditions for entry 2)

TMSOTf (6.3 μL, 35 μmol) was added to a mixture of the solid-supported acceptor **14** (28.7 μmol), donor **2** (101.8 mg, 70.0 μmol), and activated MS4A (beads) in CH_2Cl_2/C_4F_9OEt/THF (5:4:1) (1.0 mL) at −15 °C, and the reaction mixture was shaken at the same temperature for 1 h. After dilution and filtration with THF, the resin was washed and filtered with THF and CH_2Cl_2 (each 2 min, 3 times), CH_2Cl_2/MeOH (5:1), MeOH, CH_2Cl_2/MeOH (5:1) and CH_2Cl_2 (each 2 min, 2 times), and CH_2Cl_2 (2 min, 2 times). MS4A was removed during the resin treatment. The glycosylation yield was 83%.

To calculate the loading yield, **15** was shaken with 30% Et_3N in CH_2Cl_2 at room temperature to deprotect Fmoc groups. After the reaction was complete, the produced 9-methylene-fluorene was collected, and absorbance was measured via UV–vis spectroscopy (λ_{max} = 301 nm, ε= 7800 M·cm^{-1}).

(Conditions for entry 1)

Glycosylation was performed under conditions similar to those for entry 1. CH_2Cl_2/THF (9:1) was used as the reaction solvent. The glycosylation yield was 56%.

5-2. SPOS of octasaccharide **20**

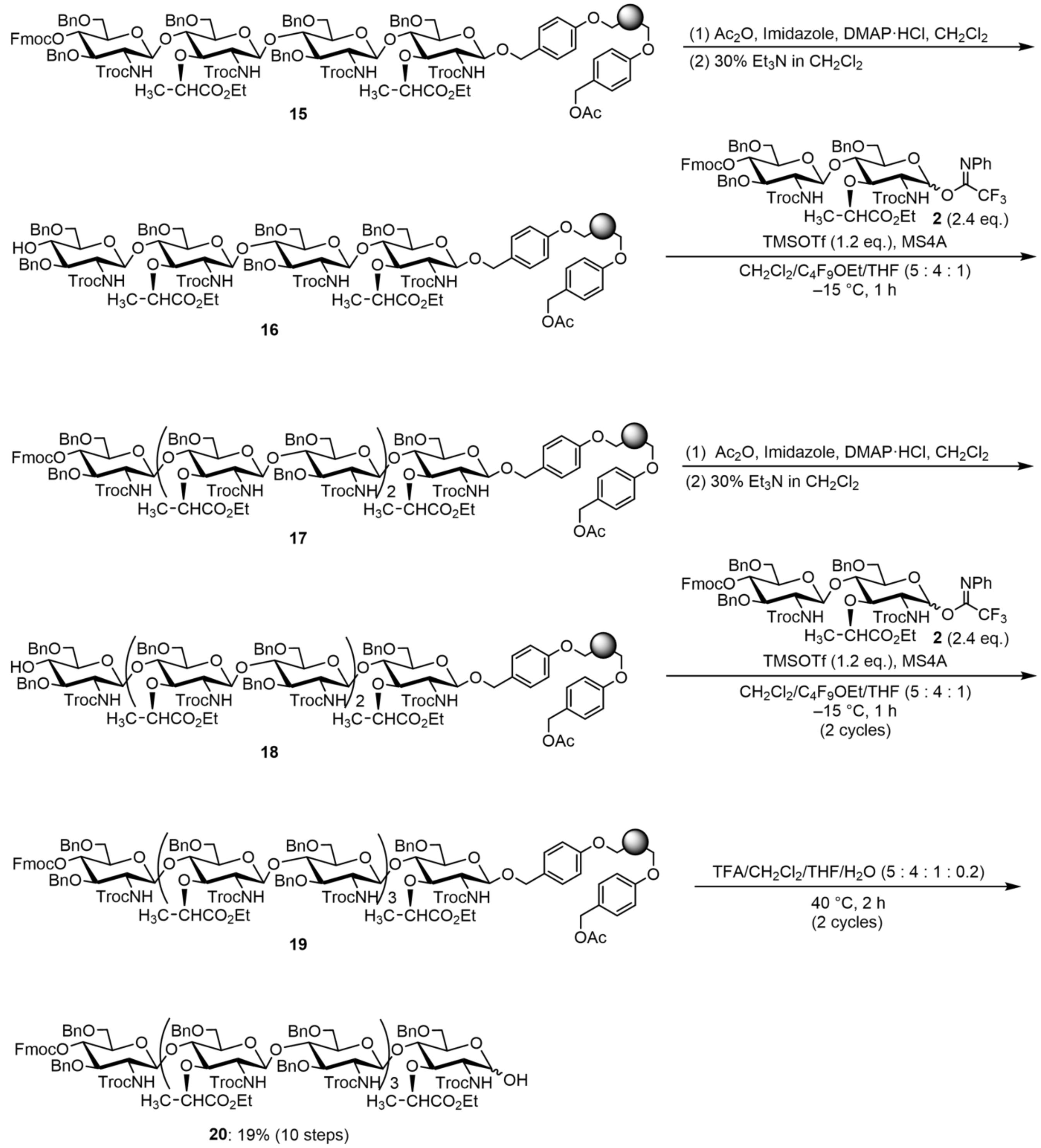

Scheme S8. SPOS of octasaccharide **20**

Solid-supported tetrasaccharide **16**

Ac_2O (908 μL, 9.60 mmol), DMAP·HCl (47.6 mg, 300 μmol), and imidazole (81.7 mg, 1.20 mmol) were added to a suspension of the solid-supported tetrasaccharide **15** (24.6 μmol) in CH_2Cl_2 (1.2 mL) at 20 °C, and the mixture was

shaken at the same temperature for 3 h. After dilution and filtration with CH_2Cl_2, the resin was washed and filtered with CH_2Cl_2 and THF (each 2 min, 4 times), CH_2Cl_2 and CH_2Cl_2/MeOH (5:1) (each 2 min, 4 times), and CH_2Cl_2 (2 min, 3 times). After washing, the resin was dried *in vacuo*.

After the resin suspension in CH_2Cl_2 was shaken for several minutes, the solvent was replaced with 30% Et_3N in CH_2Cl_2 (3.0 mL) at 20 °C, and the reaction mixture was shaken at the same temperature for 6 h. After dilution and filtration with CH_2Cl_2 (10 mL), the resin was filtered and washed with CH_2Cl_2 and THF (each 2 min, 10 times), and CH_2Cl_2 (2 min, 3 times). After washing, tetrasaccharide acceptor **16** was dried *in vacuo*.

Solid-supported hexasaccharide **17**

TMSOTf (5.0 µL, 27.5 µmol) was added to a mixture of the solid-supported acceptor **16** (22.6 µmol), donor **2** (80.0 mg, 55.0 µmol), and activated MS4A (beads) in CH_2Cl_2/C_4F_9OEt/THF (5:4:1) (1.1 mL) at −15 °C, and the reaction mixture was shaken at the same temperature for 1 h. After dilution and filtration with THF, the resin was washed and filtered with THF and CH_2Cl_2 (each 2 min, 3 times), CH_2Cl_2/MeOH (5:1), MeOH, CH_2Cl_2/MeOH (5:1) and CH_2Cl_2 (each 2 min, 2 times), and CH_2Cl_2 (2 min, 2 times). MS4A was removed during the resin treatment. The solid-supported hexasaccharide **17** was used in the subsequent reaction.

Solid-supported hexasaccharide **18**

Ac_2O (833 μL, 8.80 mmol), DMAP·HCl (43.6 mg, 275 μmol), and imidazole (74.9 mg, 1.1 mmol) were added to a suspension of hexasaccharide on resin **17** (22.6 µmol) in CH_2Cl_2 (1.1 mL) at 20 °C, and the mixture was shaken at the same temperature for 3 h. After dilution and filtration with CH_2Cl_2 (10 mL), the resin was washed and filtered with CH_2Cl_2 and THF (each 2 min, 4 times), CH_2Cl_2 and CH_2Cl_2/MeOH (5:1) (each 2 min, 4 times), and CH_2Cl_2 (2 min, 3 times). After washing, the resin was dried *in vacuo*.

After the resin suspension in CH_2Cl_2 was shaken for several minutes, the solvent was replaced with 30% Et_3N in CH_2Cl_2 (2.8 mL) at 20 °C, and the mixture was shaken at the same temperature for 6 h. After dilution and filtration with CH_2Cl_2 (10 mL), the resin was washed and filtered with CH_2Cl_2 and THF (each 2 min, 10 times) and CH_2Cl_2 (2 min, 3 times). After washing, hexasaccharide acceptor **18** was dried *in vacuo*.

Solid-supported octasaccharide **19**

TMSOTf (5.0 μL, 27.5 µmol) was added to a mixture of disaccharide acceptor **19** (22.6 µmol), donor **2** (80.0 mg, 55.0 µmol), and activated MS4A (beads) in CH_2Cl_2/ C_4F_9OEt/THF (5:4:1) (1.1 mL) at −15 °C, and the reaction mixture was shaken at the same temperature for 1 h. After dilution and filtration with THF, the resin was washed and filtered with THF and CH_2Cl_2 (each 2 min, 3 times), CH_2Cl_2/MeOH (5:1), MeOH, CH_2Cl_2/MeOH (5:1) and CH_2Cl_2 (each 2 min, 2 times), and CH_2Cl_2 (2 min, 2 times). After washing and removing MS4A, the resulting resin was dried *in vacuo*. This protocol was repeated, and the solid-supported octasaccharide **19** was obtained.

Octasaccharide **20**

After a suspension of **19** (40.0 mg, 6.15 μmol) in CH_2Cl_2 was shaken for several minutes, the solvent was replaced with TFA/CH_2Cl_2/THF/H_2O (5:4:1:0.2), and the mixture was stirred at 40 °C for 2 h. After dilution and filtration with CH_2Cl_2, the resin was washed and filtered with CH_2Cl_2 (2 min, 3 times), THF (2 min, 3 times), and CH_2Cl_2 (2 min, 3 times). The cleavage of **19** was performed using the same procedure. The filtrate of CH_2Cl_2 was collected and neutralized with aqueous $NaHCO_3$ at 0 °C. The organic layer was diluted with $CHCl_3$, washed with H_2O and brine, and dried over Na_2SO_4. After concentration, silica gel column chromatography (toluene/EtOAc, 6:1, 5:1, 3:1, and 2:1) and LH-20

column chromatography ($CHCl_3$/MeOH, 2:1) produced octasaccharide **20** (5.3 mg, 19% in 10 steps) as a white solid. The ^{1}H–NMR spectrum of **20** was consistent with that obtained via the liquid-phase synthesis.

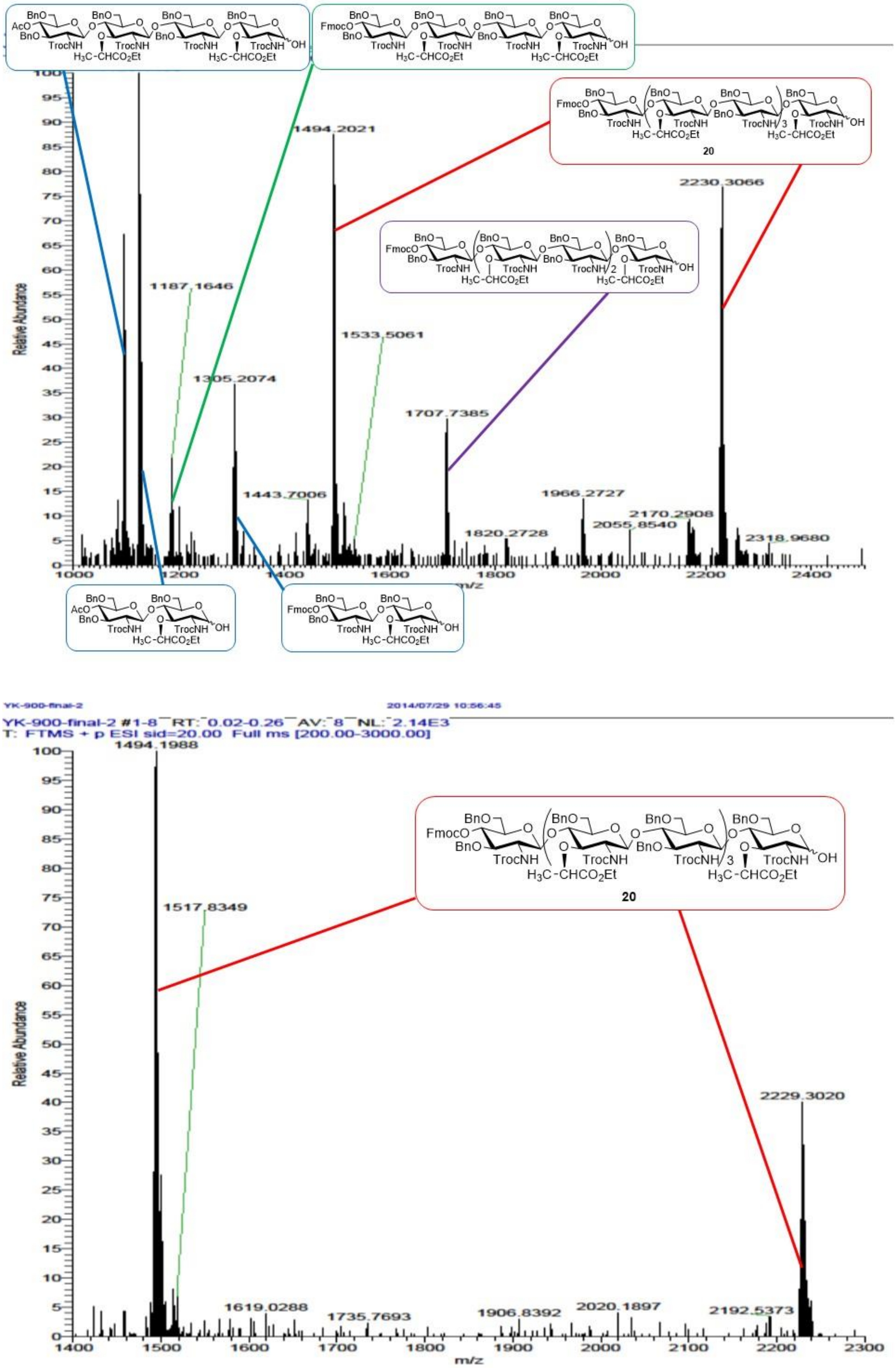

Figure S1. MS-spectra of octasaccharide **20** (top: crude, bottom: pure).

6. SPOS of octasaccharide with dipeptides 1

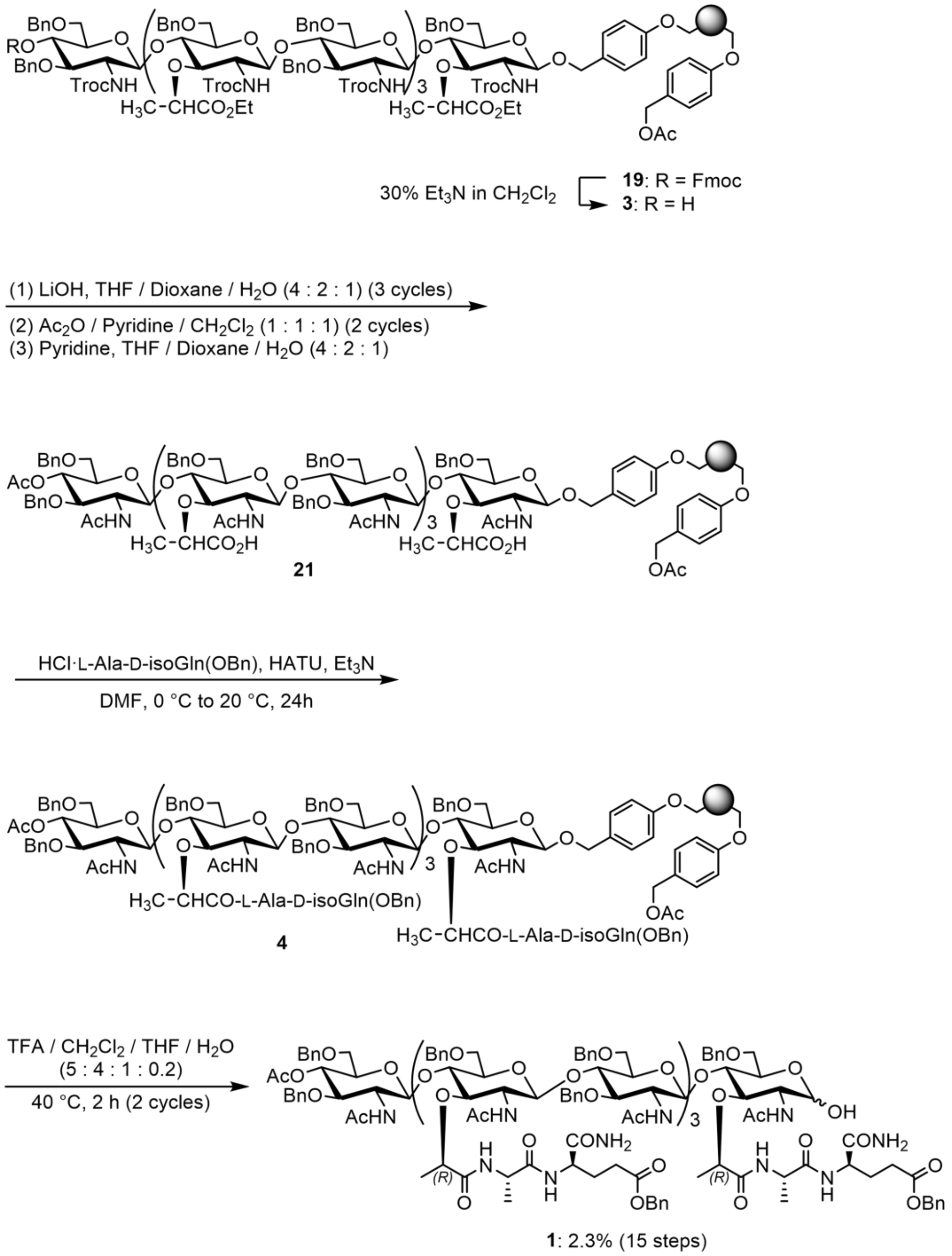

Scheme S9. SPOS of octasaccharide with dipeptides **1**.

Solid-supported octasaccharide **3**

After the suspension of **19** (6.15 μmol) in CH_2Cl_2 was shaken for several minutes, the solvent was replaced with 30% Et_3N in CH_2Cl_2 (1.0 mL) at 20 °C, and the reaction mixture was shaken at the same temperature for 6 h. After dilution and filtration with CH_2Cl_2, the resin was washed and filtered with CH_2Cl_2 (2 min, 1 time), THF (2 min, 2 times), and THF/dioxane/H_2O (4:2:1) (2 min, 2 times). Compound **3** was used in the subsequent reaction.

Solid-supported octasaccharide **21**

After a suspension of **3** (6.15 μmol) in THF/dioxane/H_2O (4:2:1) was shaken for several minutes, the solvent was replaced with $LiOH{\cdot}H_2O$ (10.1 mg, 240 μmol) in THF/dioxane/H_2O (4:2:1) (2.8 mL) at 20 °C. The reaction mixture

was shaken at the same temperature for 12 h. After dilution and filtration with THF/dioxane/H_2O (4:2:1), the resin was washed and filtered with THF/dioxane/H_2O (4:2:1) (2 min). This procedure was repeated twice. After the reaction was completed, the resin was washed and filtered with THF/dioxane/H_2O (4:2:1), dioxane/H_2O (1:1), THF/dioxane/H_2O (4:2:1) and THF (each 2 min, 3 times), and CH_2Cl_2 (2 min, 2 times). After washing, the resin was used in the subsequent reaction.

After a suspension of the resin in CH_2Cl_2 was shaken for several minutes, the solvent was replaced with Ac_2O/pyridine/CH_2Cl_2 (1:1:1) (1.5 mL) at 20 °C, and the reaction mixture was shaken at the same temperature for 12 h. This procedure was repeated after filtration. After dilution and filtration with CH_2Cl_2, the resin was washed and filtered with CH_2Cl_2 (2 min, 2 time), THF (2 min, 2 times), and THF/dioxane/H_2O (4:2:1) (2 min, 2 times). The resulting resin was used for the subsequent reaction.

Pyridine (97.0 μL, 1.2 mmol) was added to a suspension of the resin in THF/dioxane/H_2O (4:2:1) (2.8 mL) at 20 °C, and the reaction mixture was shaken at the same temperature for 24 h. After dilution and filtration with THF/dioxane/H_2O (4:2:1), the resin was washed and filtered with THF/dioxane/H_2O (4:2:1) (2 min, 3 times), THF (2 min, 3 times), and DMF (2 min, 3 times). The resulting compound **21** was used for the next reaction.

Solid-supported octasaccharide with dipeptides **4**

Et_3N (100 μL, 720 μmol) was added to a mixture of HCl·L-Ala-D-isoGln(OBn) (123.8 mg, 360.0 μmol), HATU (136.9 mg, 360.0 μmol), and the solid-supported **21** (6.15 μmol) in DMF (1.5 mL) at 0 °C, and the reaction mixture was shaken at 20 °C for 24 h. After dilution and filtration with DMF (10 mL), the resin was washed and filtered with DMF (2 min, 2 times), THF (2 min, 2 times), and CH_2Cl_2 (2 min, 3 times). The resulting compound **4** was used in the subsequent reaction.

Octasaccharide with dipeptides **1**

After the suspension of **4** (6.15 μmol) in CH_2Cl_2 was shaken for several minutes, the solvent was replaced with TFA/CH_2Cl_2/THF/H_2O (5:4:1:0.2), and the mixture was stirred at 40 °C for 2 h. After dilution and filtration with CH_2Cl_2, the resin was washed and filtered with CH_2Cl_2 (2 min, 3 times), THF (2 min, 3 times), and CH_2Cl_2 (2 min, 3 times). This procedure was repeated once. The filtrate of CH_2Cl_2 was neutralized with an aqueous $NaHCO_3$ solution at 0 °C and extracted with $CHCl_3$/MeOH (20:1). The organic layer was washed with H_2O and brine and dried over Na_2SO_4. After concentration, silica gel column chromatography ($CHCl_3$/MeOH = 10:1, 6:1), LH-20 column chromatography ($CHCl_3$/MeOH, 2:1) and normal-phase HPLC (Table S1) produced octasaccharide with dipeptides **1** (0.6 mg, 2% in 14 steps; average yield: >75%) as a white solid. R_f = 0.59 ($CHCl_3$/MeOH = 7:1); ^{1}H NMR (500 MHz, $CDCl_3$/CD_3OD, 10:1) δ 7.39–7.13 (m, 80 H), 5.53 (d, J = 3.2 Hz, 1H), 5.12 (s, 2H), 5.11 (s, 2H), 5.10 (s, 2H), 5.09 (s, 2H), 5.05 (t, J = 9.7 Hz, 1H), 4.88 (d, J = 11.3 Hz, 1H), 4.86 (d, J = 12.2 Hz, 1H), 4.71–4.64 (m, 1H), 4.66 (d, J = 11.9 Hz, 1H), 4.62–4.32 (m, 30H), 4.58 (t, J = 8.8 Hz, 1H), 4.35 (d, J = 8.7 Hz, 1H), 4.34 (d, J = 7.9 Hz, 1H), 4.32–4.18 (m, 12 H), 4.23 (d, J = 7.0 Hz, 1H), 4.19 (d, J = 7.2 Hz, 1H), 4.04 (q, J = 6.8 Hz, 2H), 4.00–3.91 (m, 5H), 3.91–2.99 (m, 36H), 2.54–2.29 (m, 8H), 2.24–2.14 (m, 8H), 1.98 (s, 3H), 1.95 (s, 3H), 1.91 (s, 3H), 1.90 (s, 3H), 1.89 (s, 3H), 1.81 (s, 3H), 1.79 (s, 3H) 1.74 (s, 3H), 1.74 (s, 3H), 1.51–1.05 (m, 24H); HRMS (ESI-LIT Orbitrap) calcd. for $C_{222}H_{272}N_{20}O_{62}Na_3$ $[M+3Na]^{3+}$ 1426.2808, found 1426.2811.

Table S1

Time (min.)	B
0.01-5.00	6 to 16%
5.00-8.00	16%
8.00-9.00	16 to 6%
9.00-12.00	6%

HPLC Column: Cosmosil 5SL-II (4.6ID * 250mm)
A solvent: CHCl3; B solvent: MeOH. Linear gradient.
Reteition time of **1**: approximately 9.17 min.

Table S1. HPLC conditions for the purification of **1**.

7. Synthesis of deprotected octasaccharide with dipeptides S8

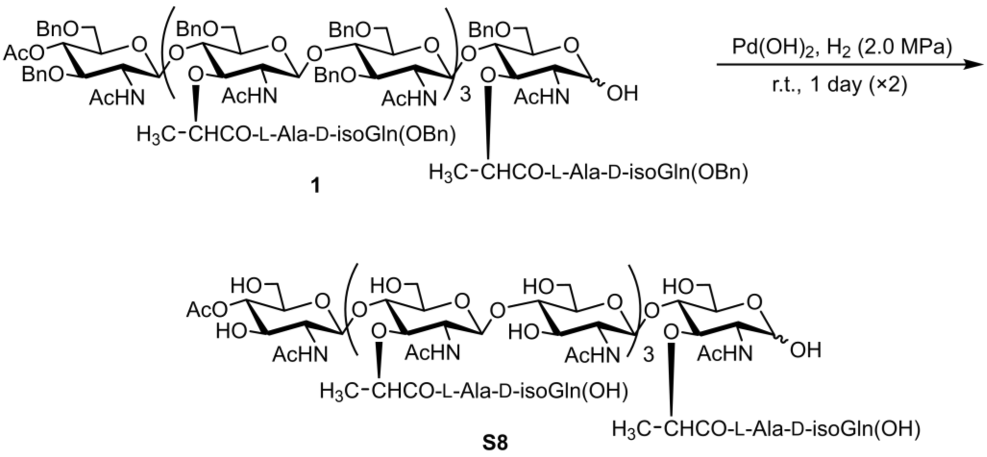

HRMS (ESI-LIT-Orbitrap) calcd. for $C_{110}H_{176}N_{20}O_{62}Na_2$ $[M+2Na]^{2+}$ 1407.5509, found 1407.5516.

Scheme S10. Synthesis of octasaccharide with dipeptides **S8**.

Pd/$(OH)_2$ was added to a solution of **1** (a small amount) in AcHO, and hydrogenation was performed under a H_2 atmosphere (2.0 MPa). After the celite filtration of the reaction mixture with H_2O, the crude product was evaporated. The HRMS spectrum reveals a molecular weight of **S8**. HRMS (ESI-LIT Orbitrap) calcd. for $C_{110}H_{176}N_{20}O_{62}Na_2$ $[M+2Na]^{2+}$ 1407.5509, found 1407.5516.

8. ^{1}H- and ^{13}C NMR spectra

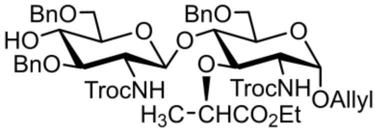

S2

^{1}H-NMR (500 MHz)

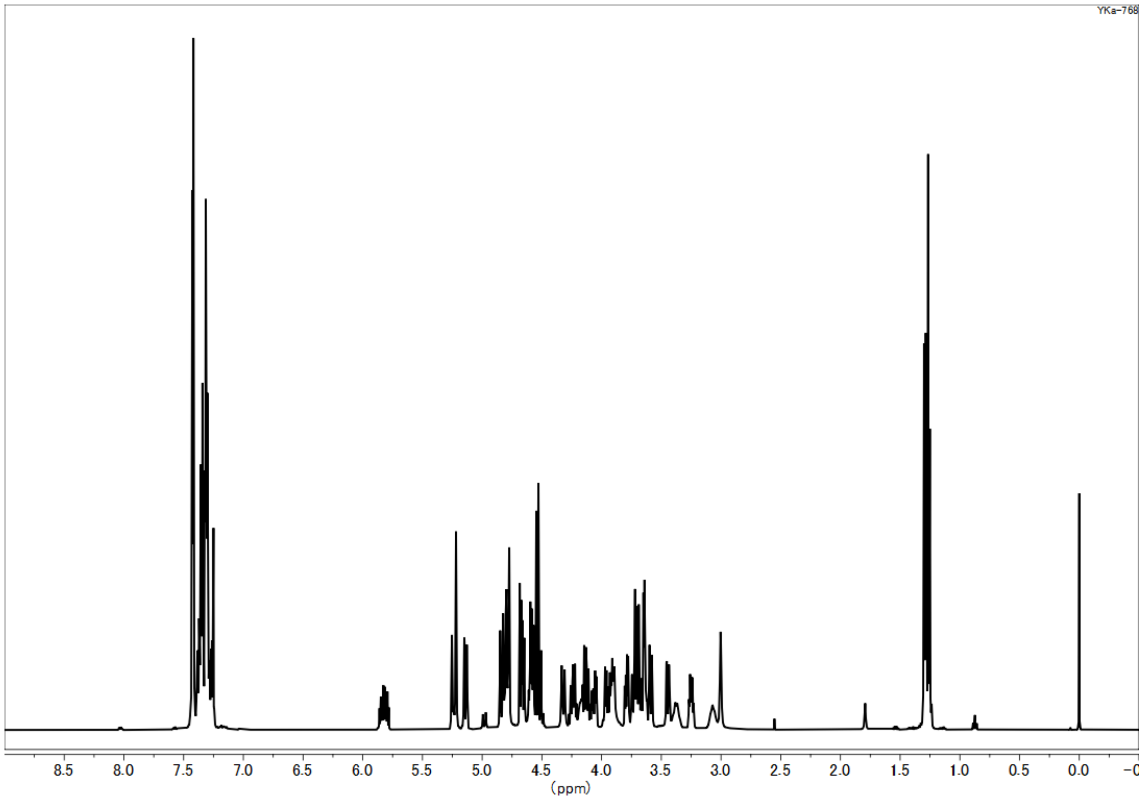

^{13}C-NMR (500 MHz)

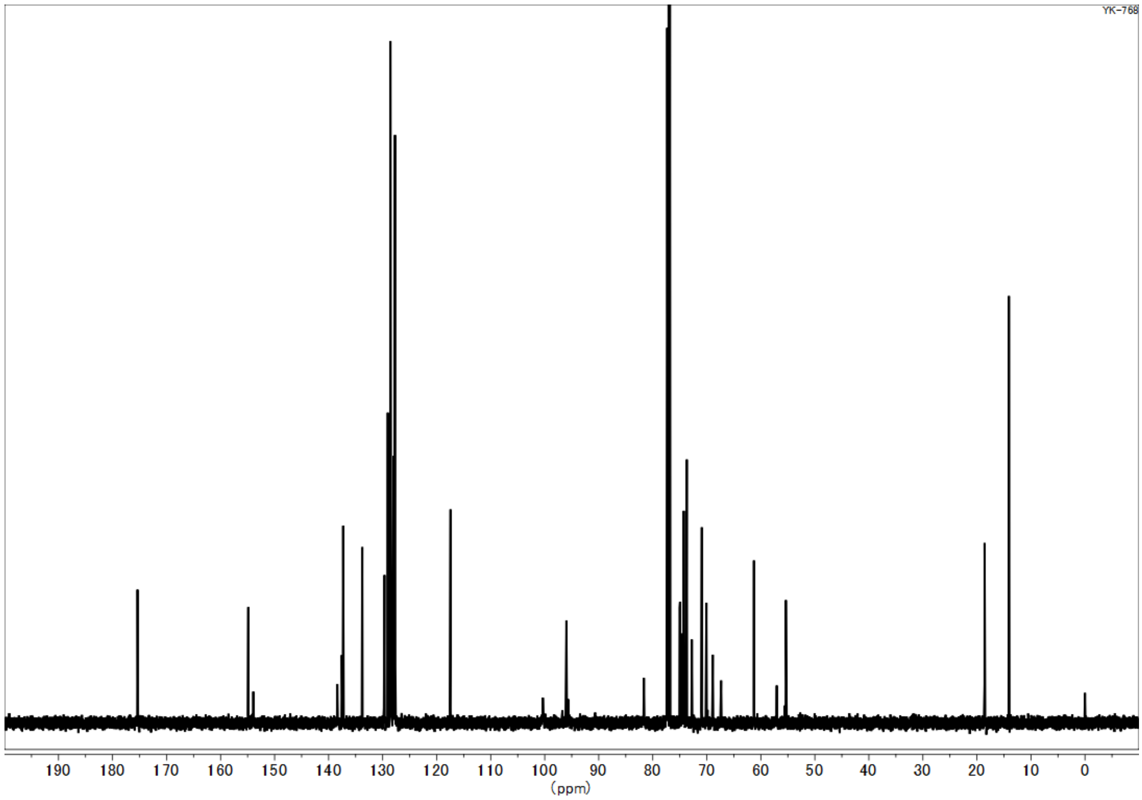

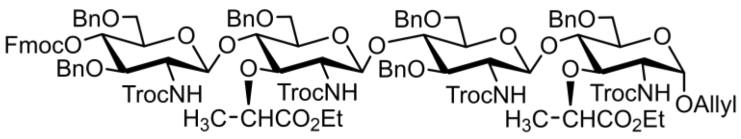

S3

^{1}H-NMR (500 MHz)

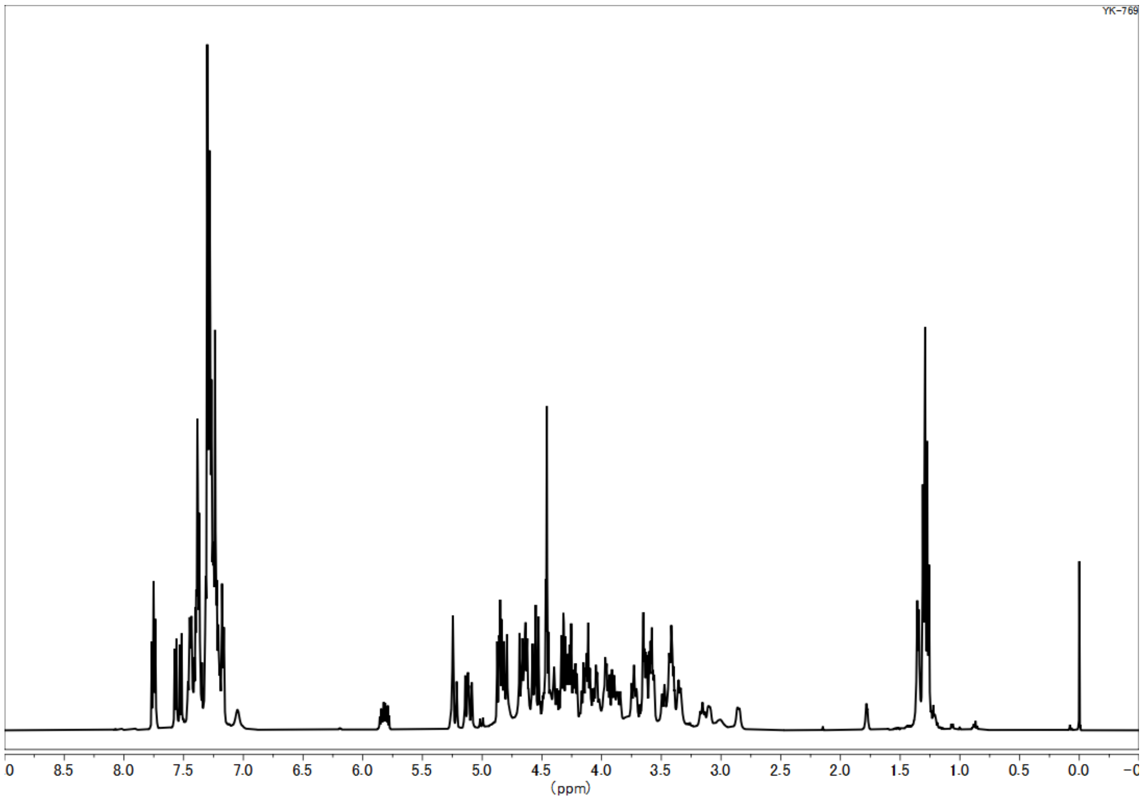

^{13}C-NMR (500 MHz)

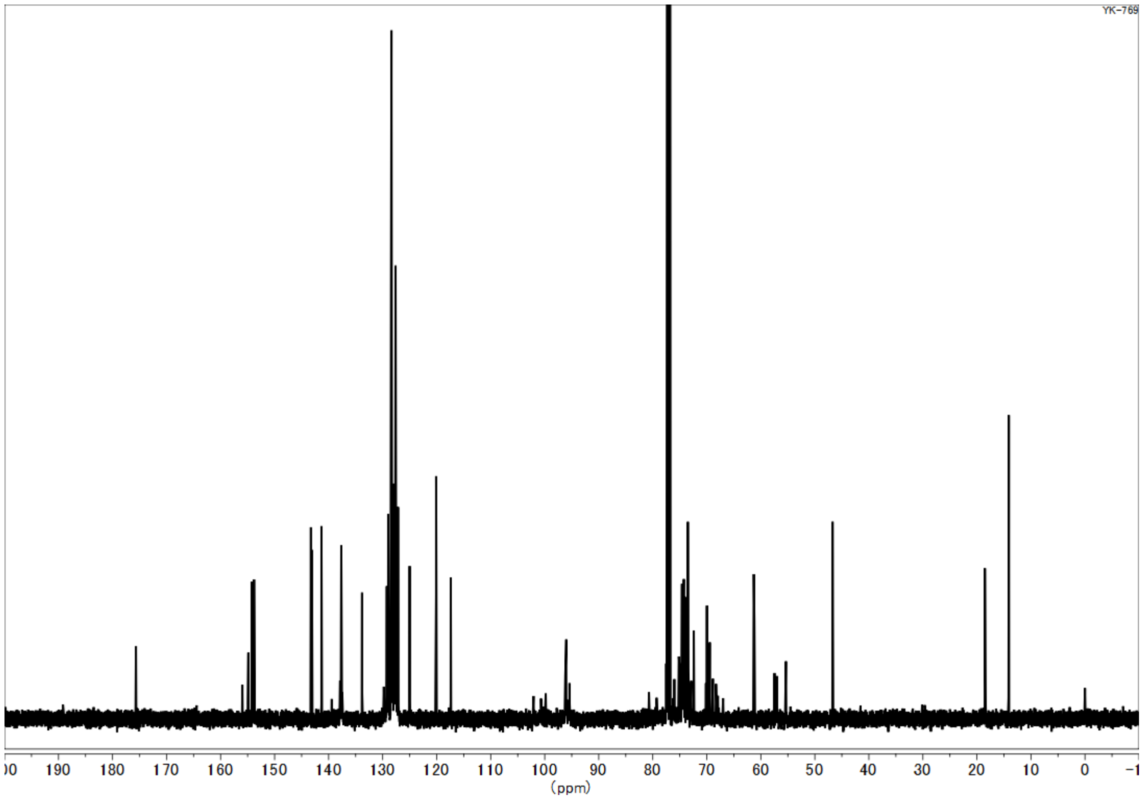

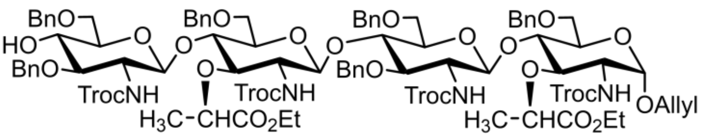

S4

^{1}H-NMR (500 MHz)

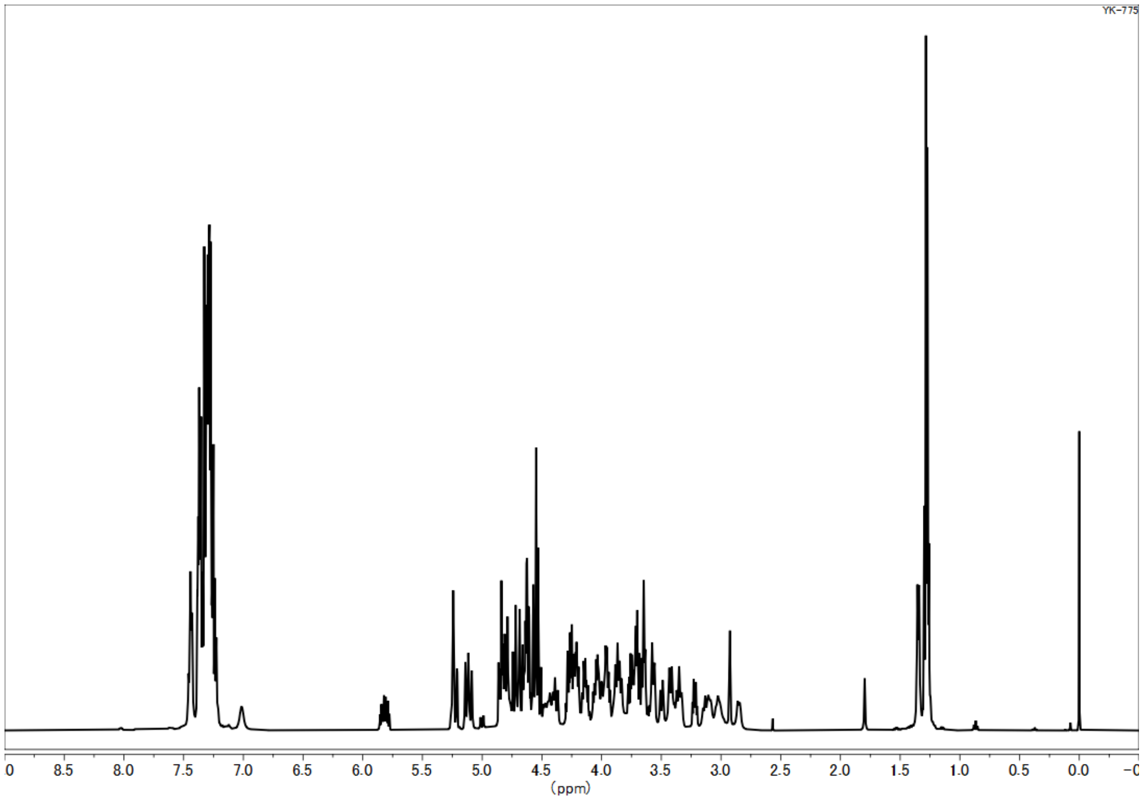

^{13}C-NMR (500 MHz)

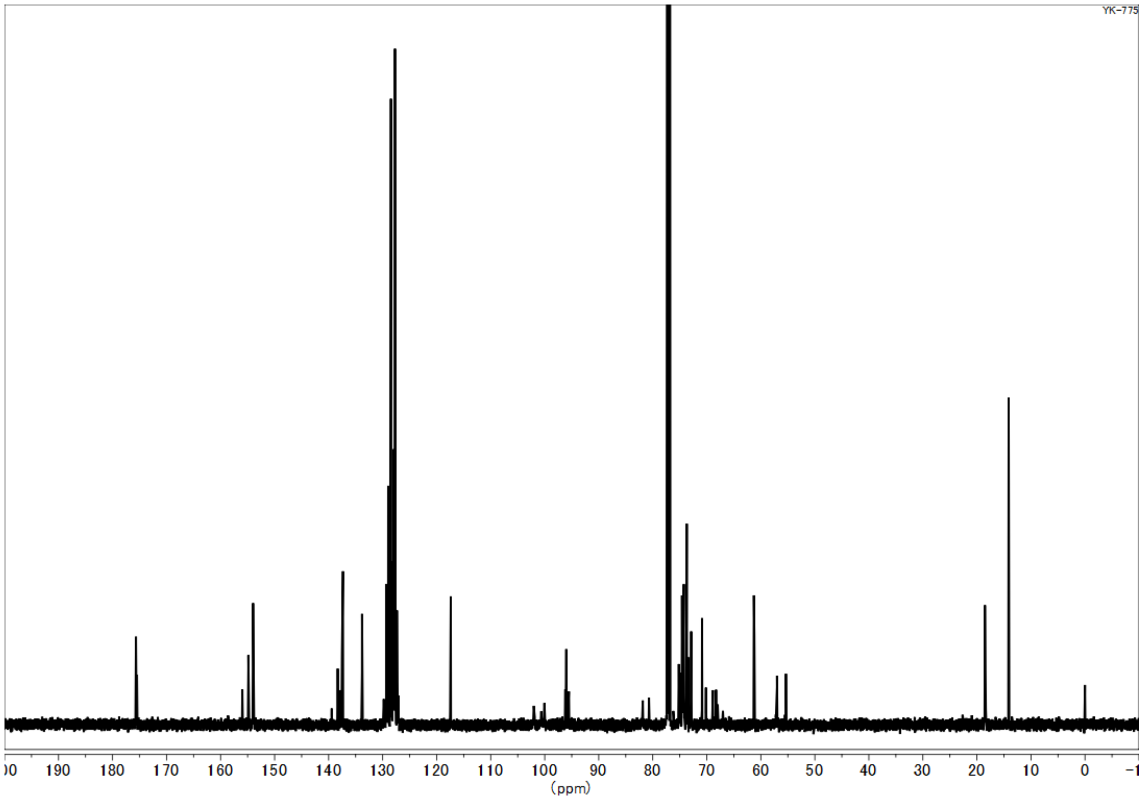

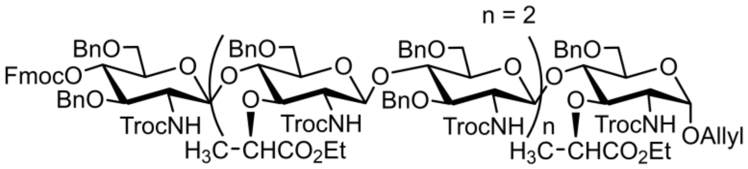

S5

^{1}H-NMR (500 MHz)

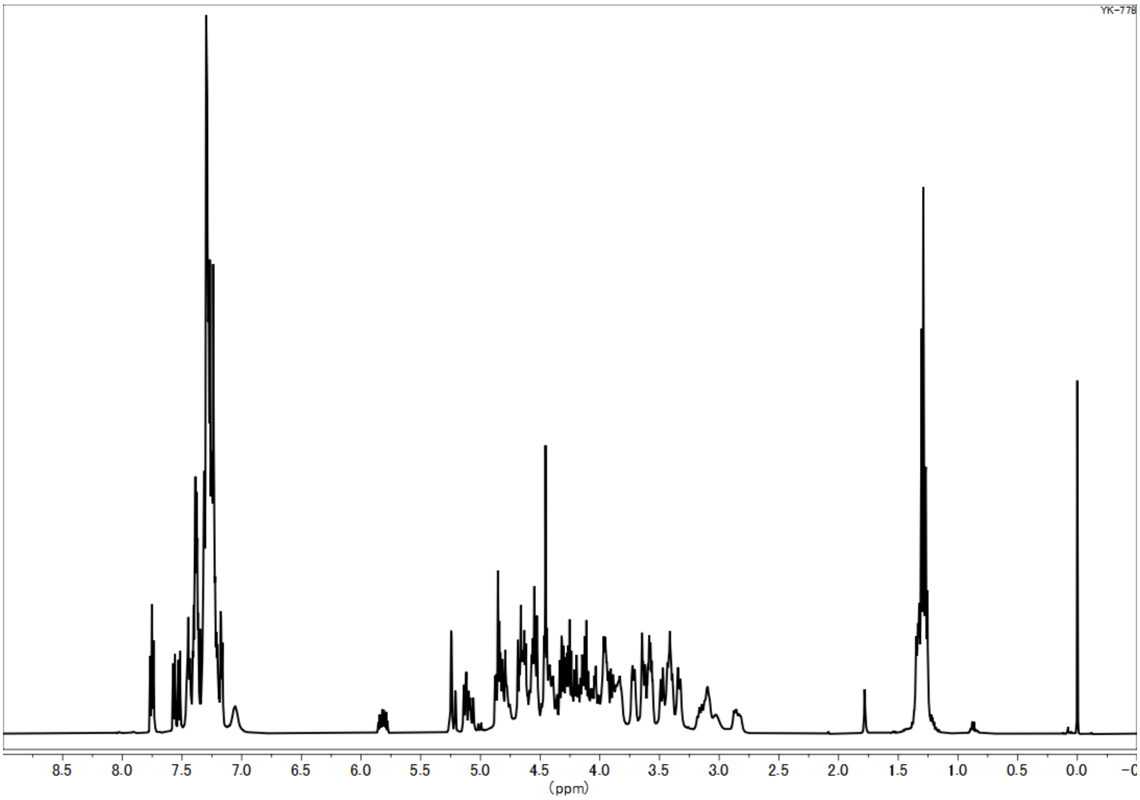

^{13}C-NMR (500 MHz)

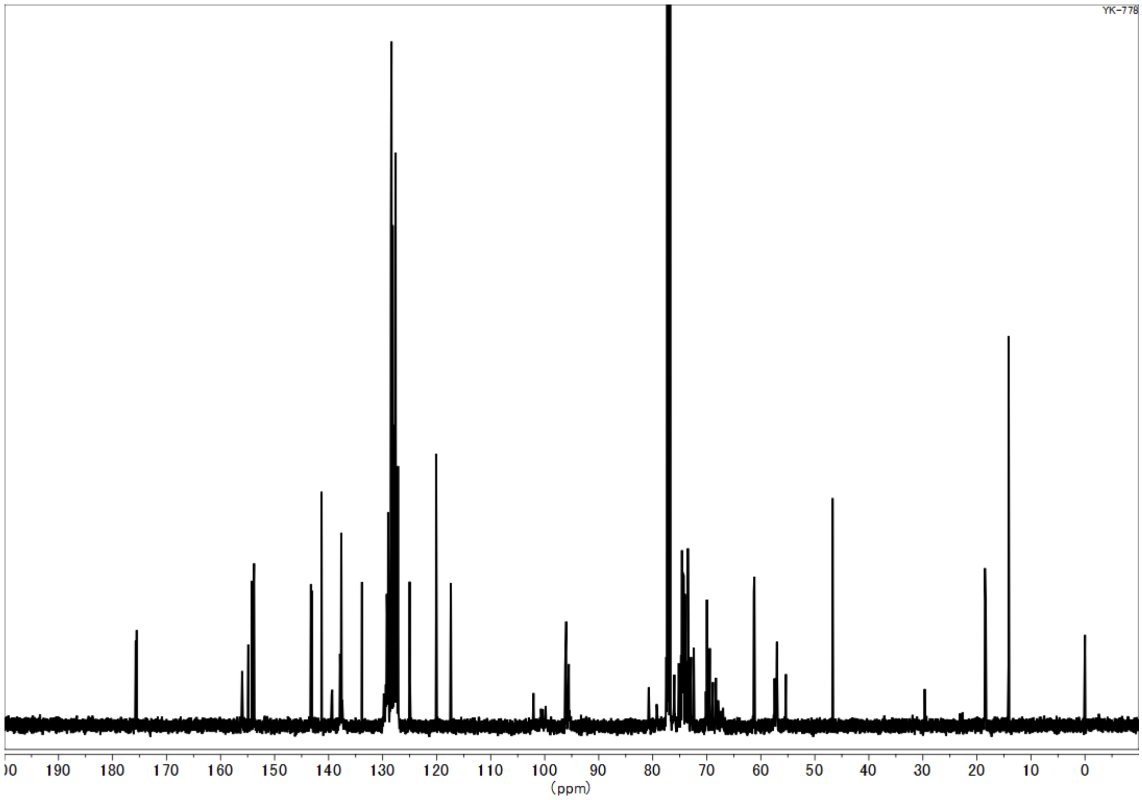

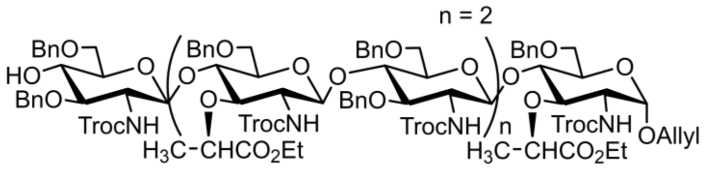

S6

^{1}H-NMR (500 MHz)

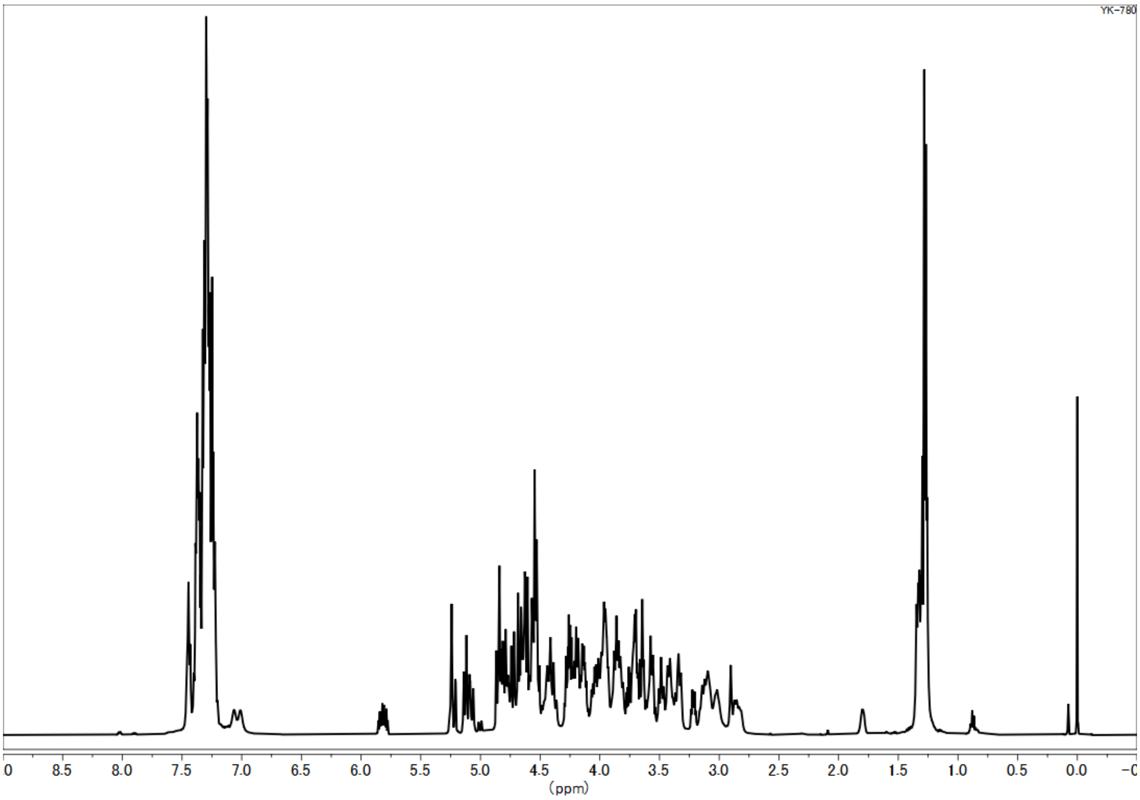

^{13}C-NMR (500 MHz)

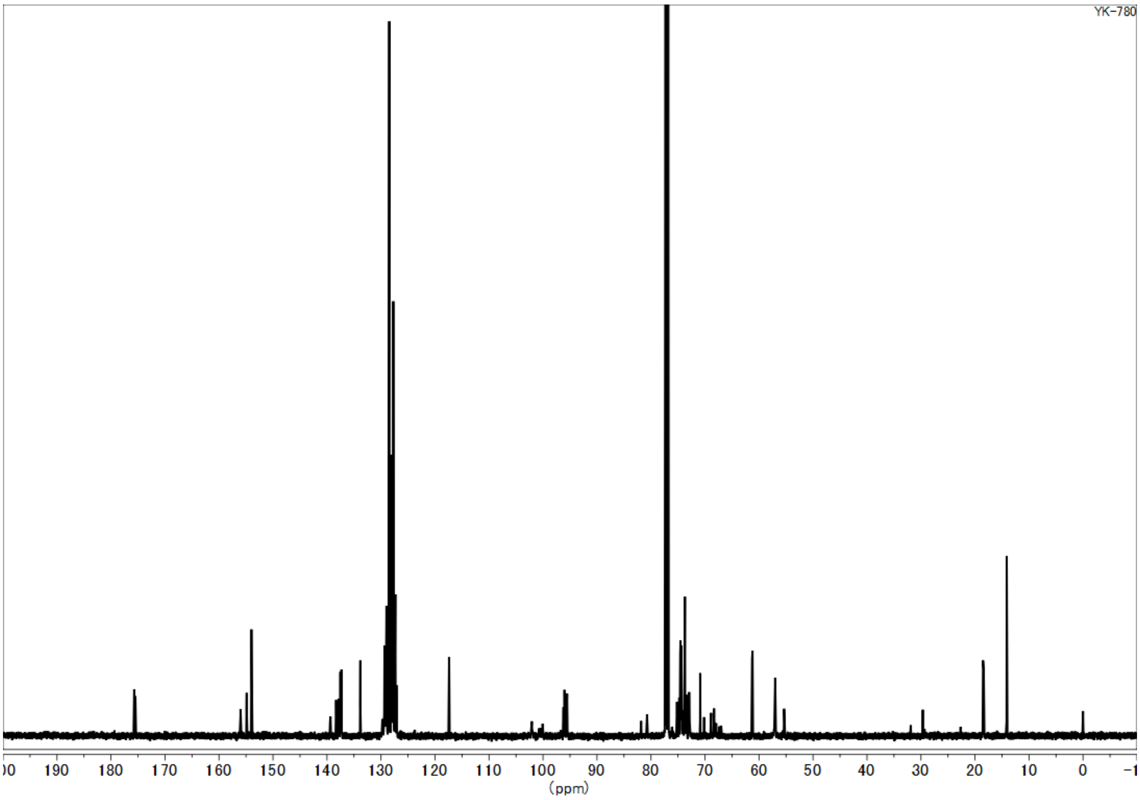

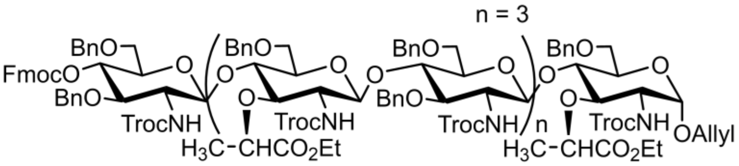

S7

^{1}H-NMR (500 MHz)

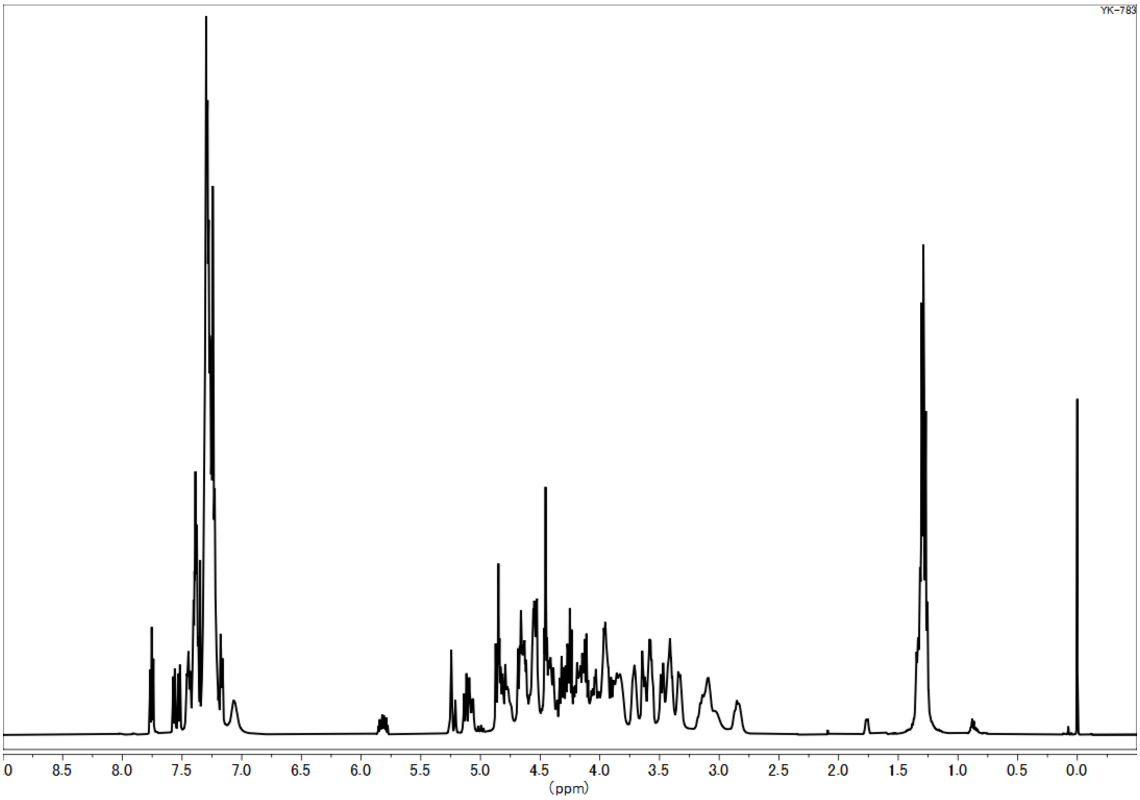

^{13}C-NMR (500 MHz)

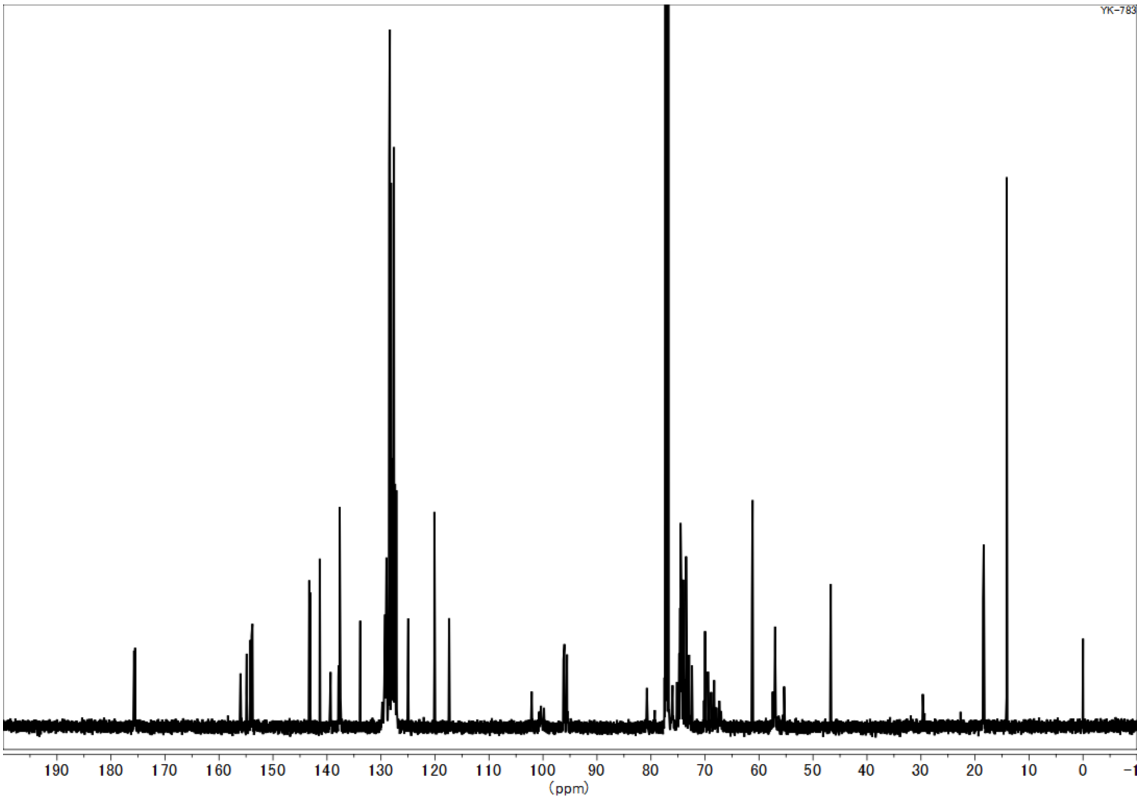

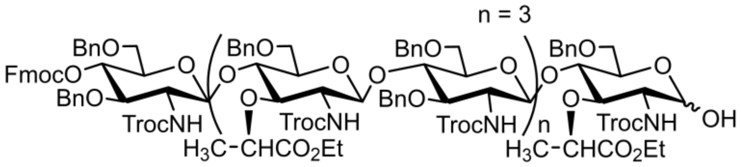

20

^{1}H-NMR (700 MHz)

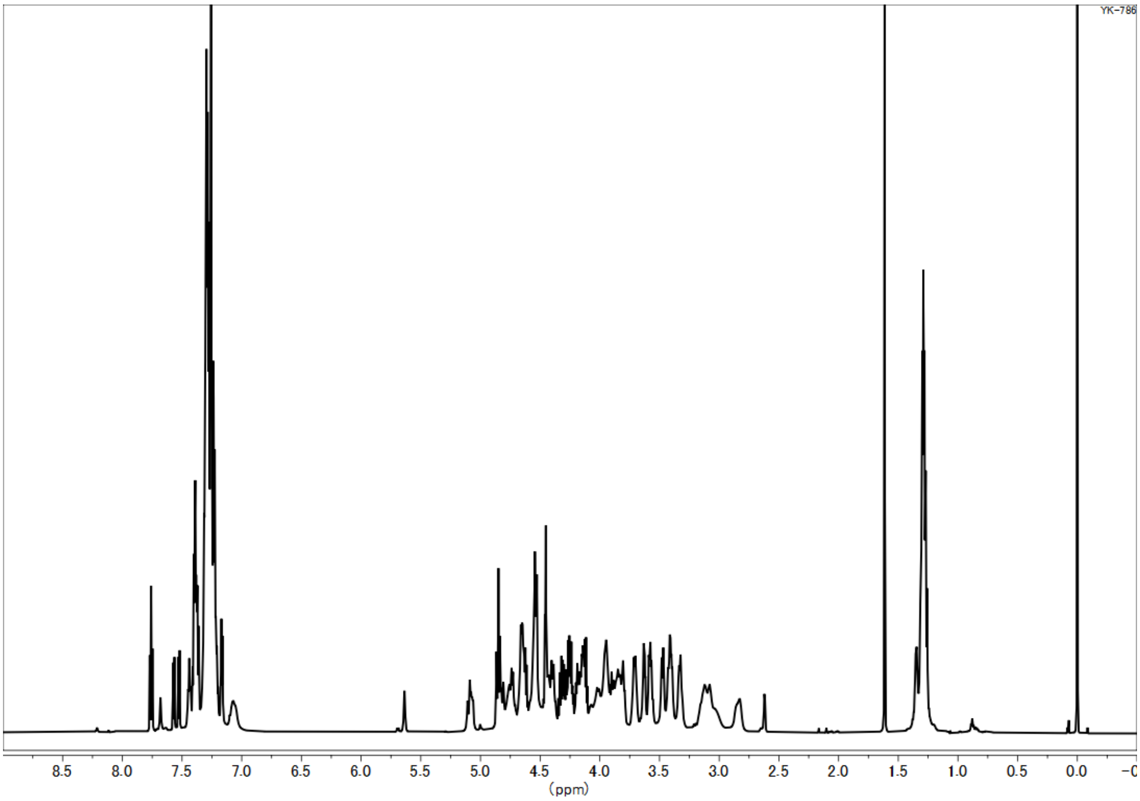

^{13}C-NMR (700 MHz)

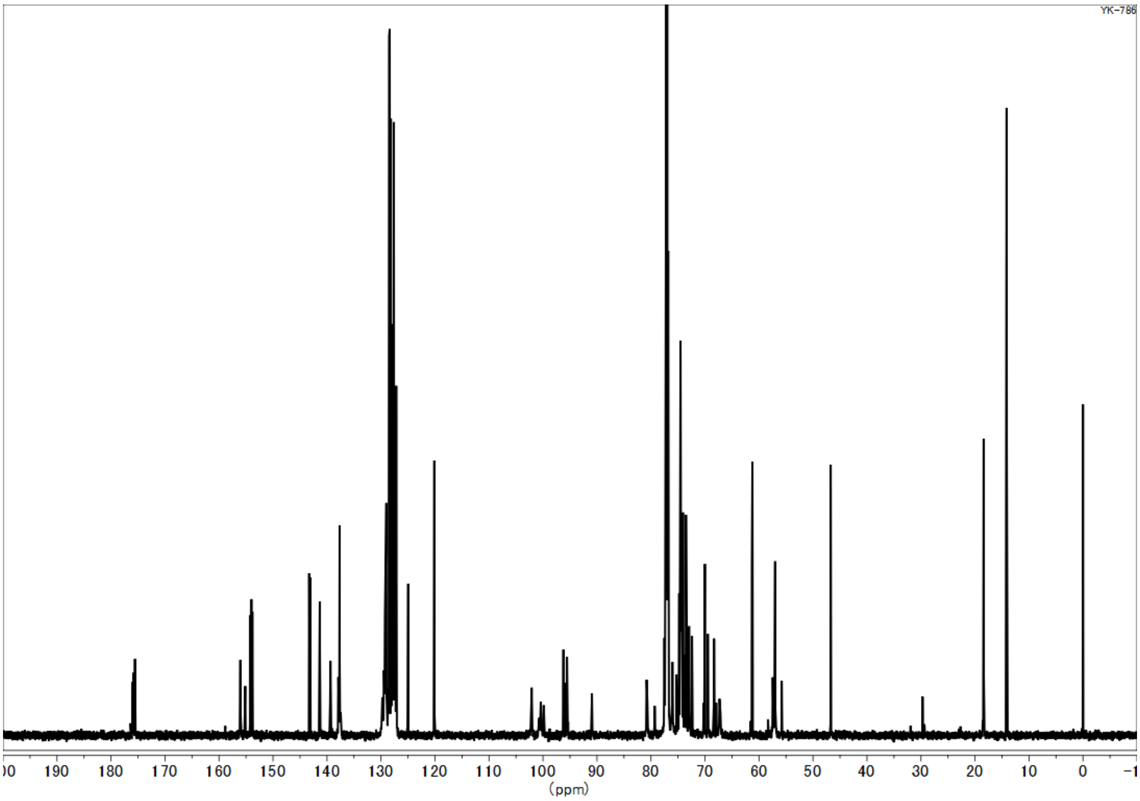

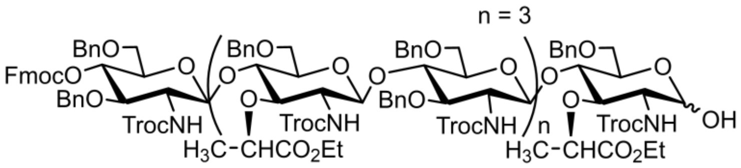

20

^{1}H-NMR (500 MHz), Prepared by liquid-phase synthesis

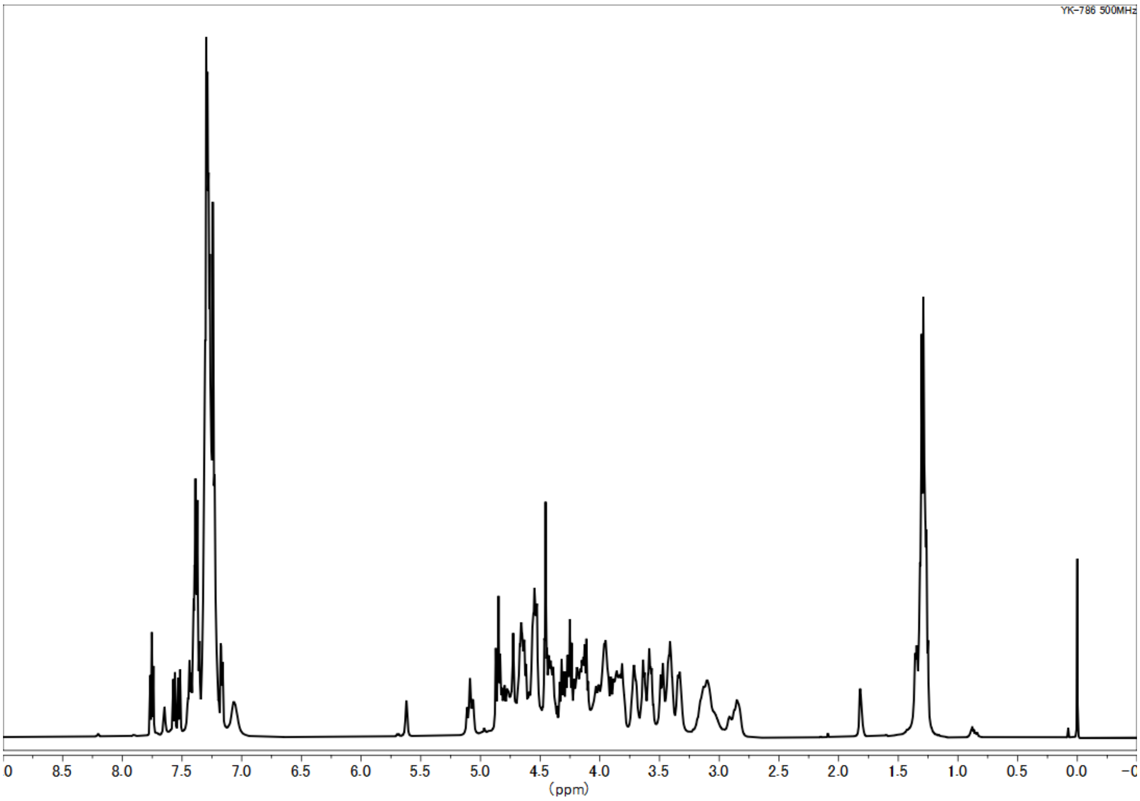

^{1}H-NMR (500 MHz), Prepared by solid-phase synthesis

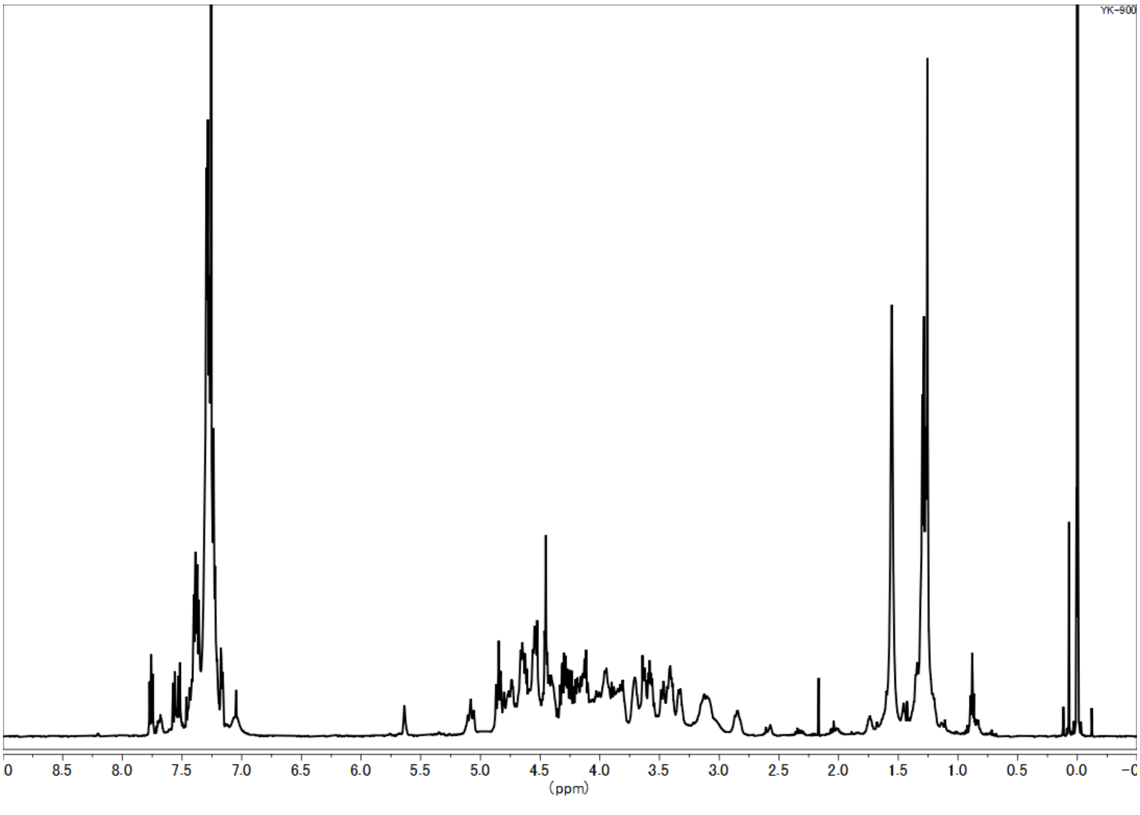

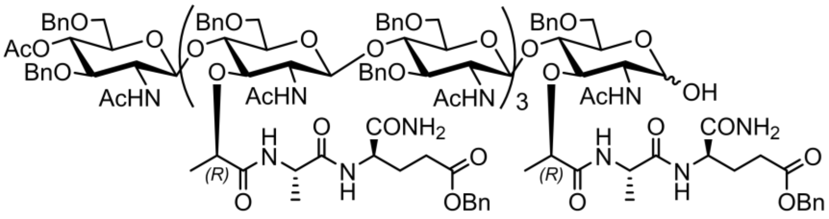

1

^{1}H-NMR (500 MHz)

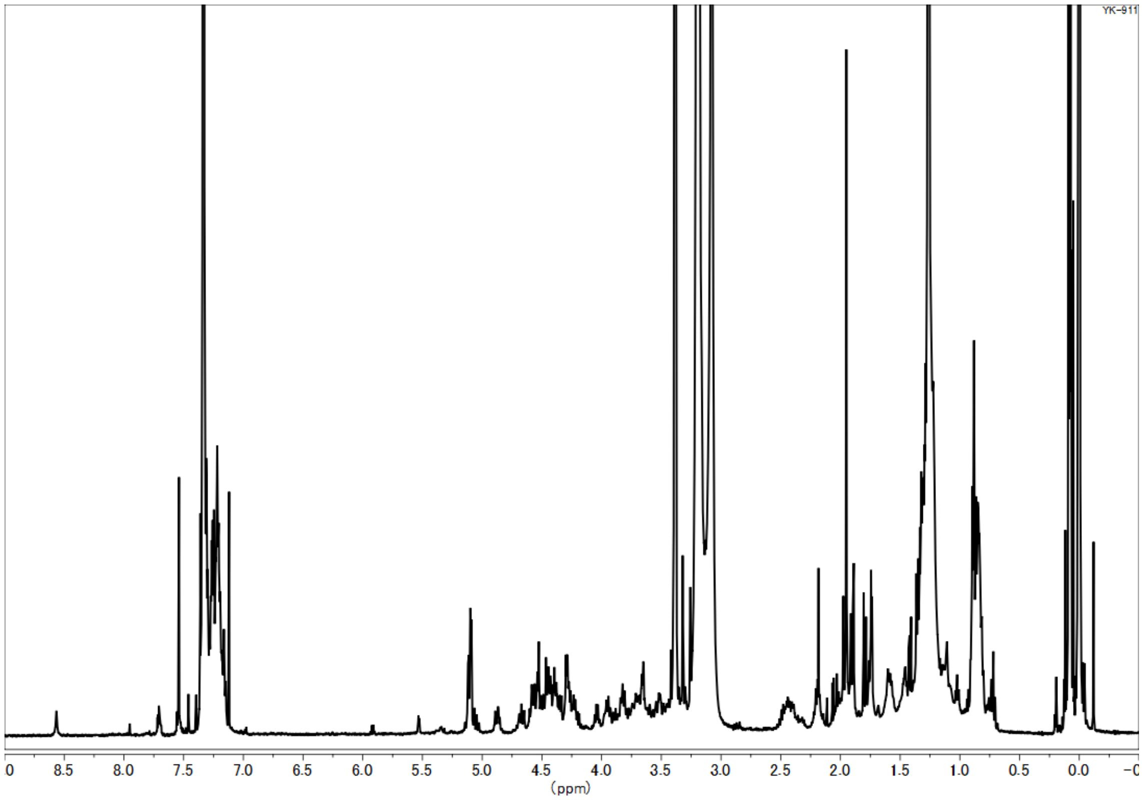

Supplement: Supplementary file 1 [file molecules-30-02787-s001.zip › molecules-3712544-supplementary materials.pdf]
